# Design, synthesis, and analysis of multi-layered 3D fluorescent polymers derived from anthracene and naphthalene structural units

**DOI:** 10.1039/d5ra08369a

**Published:** 2026-01-15

**Authors:** Hao Liu, Zacheaus M. Akinpelu, My Phan, Qingkai Yuan, Bijin Li, Delgado Cordoba Lina, Hariz Nawaz, Anthony F. Cozzolino, Dimitri Pappas, Guigen Li

**Affiliations:** a Department of Chemistry and Biochemistry, Texas Tech University Lubbock Texas 79409 USA guigen.li@ttu.edu; b School of Pharmaceutical Sciences, Chongqing University Chongqing 401331 P. R. China

## Abstract

A new class of polymers integrating anthracene and naphthalene was designed and synthesized. The materials adopt compact multilayered architectures that exploit extended π-conjugation and the rigidity of polycyclic aromatic hydrocarbon units. Optimized Suzuki–Miyaura polycondensation afforded polymers characterized by ^1^H/^13^C NMR and gel permeation chromatography (GPC). Transmission electron microscopy (TEM) images reveal nanoscale domains with locally lamellar-like contrast features in selected regions, consistent with short-range ordering rather than uniform long-range periodicity across the entire sample. Thermogravimetric analysis (TGA) and differential scanning calorimetry (DSC) indicate decomposition above 250 °C and glass-transition behavior typical of thermally robust aromatic polymers. UV-vis absorption revealed strong π–π* transitions in the UV; steady-state fluorescence displayed pronounced aggregation-induced emission (AIE) in the aggregated state. Dynamic light scattering (DLS) yielded intensity-weighted apparent hydrodynamic diameters from the nano-to sub-micrometer range with sample-dependent mono- or bimodal distributions, consistent with aggregate-rich dispersions that accompany the AIE response. Scanning electron microscopy (SEM) suggests layered surface textures without obvious macroscopic phase separation. Density-functional calculations indicate a layer-dependent shift toward more uniform π-delocalization; monomer sequence and layer number modulated the HOMO–LUMO separation. Overall, these multilayer 3D polymers combine strong luminescence, thermal stability, and tunable structure-photophysics, suggesting potential for optoelectronic and sensing applications.

## Abstract 2

New anthracene–naphthalene multilayer polymers from Suzuki polycondensation of tailored dibromonaphthalenes show water-triggered AIE turn-on and locally ordered nanoscale domains for optical sensing and optoelectronics.

## Graphical abstract

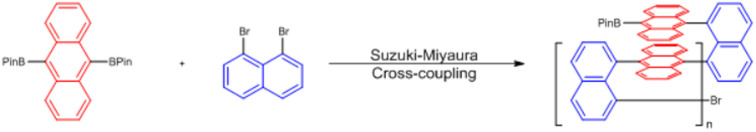

## 1 Introduction

There has been growing interest in polycyclic aromatic hydrocarbons (PAHs) as potential monomers for the creation of novel polymer materials,^1–3^ especially anthracene and naphthalene.^4–9^ These compounds are characterized by rigid, π-extended scaffolds and are widely used as building blocks in conjugated polymers and luminescent materials, where their structural stability and electronic tunability enable advanced optoelectronic functions.^4,6^ In conjugated backbones, these PAH motifs can support charge-transport behavior, while their rigid aromatic cores contribute to thermal robustness.^10–13^ Representative anthracene- and naphthalene-based polymers have been explored for applications ranging from organic light-emitting devices to photocuring and photoinitiated processes, illustrating the versatility of PAH units in functional polymer design.^5,8,9^

Aggregation-induced emission (AIE) has become a particularly powerful concept for overcoming aggregation-caused quenching in planar aromatic chromophores.^7^ A number of PAH-based luminogens and polymer systems exhibiting AIE or AIE-related behavior have been reported, including anthracene- and naphthalene-containing architectures in which emission is enhanced in the aggregated or solid state.^7,9^ In many of these studies, the choice of PAH core and side-chain substitution is used to restrict intramolecular motions and to tune intermolecular packing, thereby regulating the balance between radiative and nonradiative decay pathways. However, most of the reported PAH-based AIE systems are discrete molecules, linear polymers, or effectively two-dimensional networks, and their three-dimensional connectivity is generally not designed to probe layer-by-layer effects on photophysical properties.

Despite extensive advances in two-dimensional polymers and in three-dimensional covalent organic frameworks, the systematic integration of anthracene/naphthalene motifs into dense, non-reticular multilayer three-dimensional (3D) polymer networks remains underexplored.^14,15^ Previous work on COFs and related extended frameworks has demonstrated that precise control over connectivity and topology can be used to engineer electronic and optical properties,^14,15^ but these systems differ fundamentally from the non-porous, solution-processable polymers targeted here. In particular, there are few examples that explicitly connect (i) multilayer PAH architectures, (ii) monomer sequence, and (iii) peripheral substitution to the resulting aggregation behavior, AIE response, and thermal stability in dense PAH-based 3D polymers.

Accordingly, this work pursues three objectives: (i) design and synthesis of multilayer 3D polymers that incorporate anthracene and naphthalene; (ii) comprehensive physicochemical characterization, including molecular-weight analysis, optical absorption and fluorescence (with an emphasis on AIE behavior), thermal analysis, and morphology; and (iii) elucidation of how multilayer architecture, monomer sequence, and substituent pattern regulate aggregation-induced emission and related electronic structure. By comparing the optical and thermal properties of our multilayer polymers with those of previously reported PAH-based systems,^4,7,9^ we aim to refine structure–property principles for PAH-based 3D polymer systems and to inform materials development for electronics, optoelectronics, and sensing.^16^

Guided by these objectives, we chose to keep a rigid anthracene–naphthalene π-framework and to systematically vary the substituents on the naphthalene unit. By introducing alkyl groups at the 5,6-positions and alkoxy groups at the 2,7-positions, we aim to improve and fine-tune solubility, adjust steric hindrance and intermolecular packing, and examine how these changes influence aggregation-induced emission (AIE) and multilayer stacking. This design forms the basis for the structure–property relationships that we discuss for polymers 1A–6A in the following sections.

## 2 Results and discussion

In this work, multilayer polymer architectures were constructed by Suzuki–Miyaura polycondensation between anthracene-9,10-bis(pinacolboronate) and 1,8-dibromonaphthalene derivatives. This monomer pairing furnishes π-extended backbones while preserving the rigidity of the PAH units.

### 2.1 Synthesis and structure analysis

Anthracene-9,10-bis(pinacolboronate) monomer A was synthesized from 9,10-dibromoanthracene through the Miyaura borylation reaction in 81% isolated yield.^17^

**Fig. 1:**
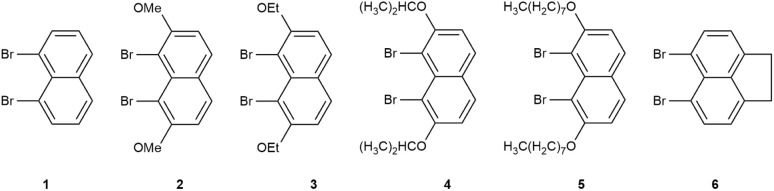
Structures of monomers 1–6.

To increase the structural diversity on the naphthalene unit, monomers 2–5 were synthesized in two steps from naphthalene-2,7-diol (2,7-dihydroxynaphthalene): *O*-alkylation to install solubilizing alkyl/alkoxy substituents at the 2,7-positions, followed by electrophilic bromination to afford the corresponding 1,8-dibromonaphthalene derivatives. Monomer 6 was prepared by electrophilic bromination of a commercially available precursor. Overall, monomers 2–6 were obtained in 63–83% yield (Fig. 1).^7,11,18,19^ The identities of all precursors were verified by NMR spectroscopy and HRMS; complete characterization data are collected in the SI.

To link monomer structure with the properties of the resulting polymers, we briefly consider the substituent effects in naphthalene derivatives 2–6 relative to the parent naphthalene framework. The introduced alkyl and alkoxy chains are electron-donating groups, which can slightly enrich the electron density on the aromatic core and subtly modulate the local π-conjugation. More importantly, increasing chain length and, in some cases, branching increases the steric demand around the peri (1,8) region of the naphthalene core while improving solubility. This balance is expected to mitigate excessively tight π–π contacts yet still permit ordered aggregation, often associated with aggregation-induced emission (AIE) and multilayer packing in the corresponding polymers.^7,19^ Consistent with common observations in side-chain engineering of π-conjugated polymers, such steric/solubility tuning can also influence polymerization efficiency and interchain organization; we therefore examine how these substituent variations translate into the molecular-weight distributions, photophysical responses, and thermal behavior of 1A–6A in the following sections.

**Fig. 2:**
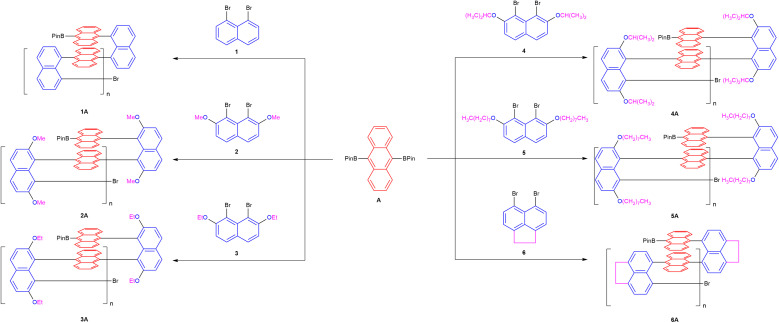
The synthetic polymers 1A–6A.

To optimize polymerization conditions, we selected 1A as a model system to optimize the Suzuki–Miyaura polycondensation. Screening several palladium–ligand complexes and solvents led us to identify tetrakis(triphenylphosphine)palladium(0) [Pd(PPh_3_)_4_] as the most effective catalyst, potassium acetate (KOAc) as the base, and a THF/H_2_O (5 : 1) mixture as the co-solvent (Table 1). The general polymerization of monomers 1–6 with monomer A to give polymers 1A–6A under these conditions is shown in Fig. 2.

**Table 1:** Optimization of the Suzuki condensation polymerization conditions

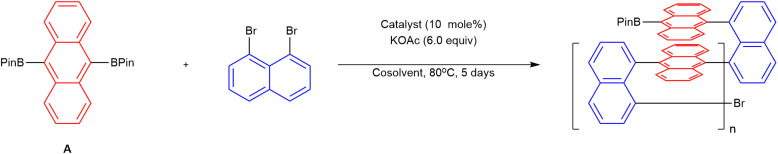				
Entry	Catalyst	Base	Solvent	Yield^a^ (%)
1	Pd(dppf)Cl_2_	KOAc	THF/H_2_O (5 : 1)	5%
2	Pd(OAc)_2_	KOAc	THF/H_2_O (5 : 1)	46%
3	Pd(PPh_3_)_2_Cl_2_	KOAc	THF/H_2_O (5 : 1)	30%
4	Pd(PPh_3_)_4_	KOAc	THF/H_2_O (5 : 1)	93%
5	Pd(PPh_3_)_4_	KOAc	Toluene	26%
6	Pd(PPh_3_)_4_	KOAc	Dioxane	33%

a Isolated yield based on substrate 1 and A.

Six different comonomer pairings were evaluated, demonstrating a broad substrate scope (Fig. 2). As shown in Table 2, the polymerization reactions afforded 1A–6A, in yields ranging from 51% to 93%.

**Table 2:** Results of synthetic polymers

Entry	Polymer	Yield^a^ (%)	*M*_n_ b	*M*_w_ b	PDI^c^
1	1A	93%	6619	7254	1.096
2	2A	51%	6391	6729	1.053
3	3A	54%	6062	6725	1.109
4	4A	51%	6736	7226	1.073
5	5A	85%	7108	7637	1.078
6	6A	86%	7283	7736	1.062

a Isolated yield based on substrate 1–6 and A.

b Determined by GPC with a polystyrene standard.

c PDI = *M*_w_/*M*_n_.

Gel Permeation Chromatography (GPC) analysis confirmed the molecular weight distributions of the synthesized polymers, the weight-average molar masses (*M*_w_) between 6725 and 7736 g mol^−1^ and the number-average molar masses (*M*_n_) from 6062 to 7283 g mol^−1^, consistent with the formation of the targeted polymer architecture (Table 2). Full analytical data and characterization reports are provided in the SI (GPC data were collected using TOSOH Eco SEC HLC-8320 and Polystyrene (PS) standards (PstQuick C) were used for calibration in our experiments).

Across polymers 1A–6A, the number-average molecular weights and dispersities show only moderate variations, but some trends with substituent type can be identified. Polymers bearing relatively short and less bulky alkoxy chains on the naphthalene unit (such as 2A) tend to exhibit narrower dispersities, which we attribute to their favorable solubility and lower steric hindrance during chain growth. In contrast, polymers derived from monomers with longer chains (*e.g.*, 4A and 5A) display somewhat broader molecular weight distributions, consistent with the increased steric congestion and more complex aggregation behavior in solution. These observations suggest that the substituent pattern on the naphthalene unit has a measurable, albeit modest, influence on the molecular weight characteristics of the resulting multilayer polymers.

The SEC/GPC traces of polymers 1A–6A show multimodal features and/or shoulders rather than perfectly monomodal distributions. This behavior is consistent with the formation of polymer populations spanning different hydrodynamic volumes and therefore indicates a broader molecular-weight distribution than would be inferred from a single peak alone. To keep the interpretation transparent, we report *M*_n_, *M*_w_, and Dstrok for each polymer and provide the full GPC traces in SI. Accordingly, we discuss the molecular-weight data in terms of overall distributions rather than assuming a single narrowly dispersed population.

### 2.2 Physicochemical characterization

**Fig. 3:**
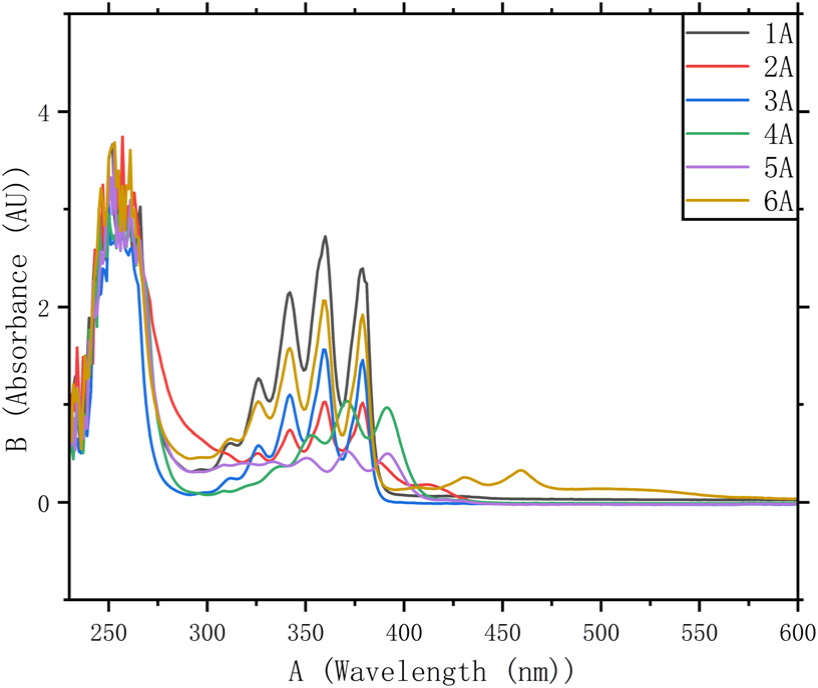
The Ultraviolet (UV) spectra of polymer 1A–6A, *c* = 0.05 mg mL^−1^ in CHCl_3_.

The UV-vis absorption spectra of 1A–6A in CHCl_3_ are shown in Fig. 3. The overlaid spectra display an intense band near ∼260 nm, along with a broad absorption band between 300 nm and 400 nm and negligible absorbance beyond ∼430–450 nm. The peaks around 250–280 nm primarily originate from the π–π* transitions of the naphthalene and anthracene units. Multiple peaks between roughly 300 nm and 400 nm, reflect the well-known *S*_0_ → *S*_1_ vibronic progression of anthracene in solution. These assignments are consistent with reference spectra and databases for PAHs.^20,21^ Above approximately 400 nm, the absorbance decreases sharply, indicating that there are no low-energy (visible-region) π–π* transitions in these polymer backbones. Overall, these naphthalene/anthracene-based polymers exhibit well-defined π–π* transitions in the UV range and maintain a rigid, conjugated backbone. The combination of strong UV absorption and potential for π-stacking makes them promising candidates for UV-blocking coatings, photonic materials, or other optoelectronic applications that require a clear cutoff in the visible region coupled with robust UV absorption.^22,23^

The influence of substituent type is more clearly reflected in the photophysical response. In dilute solution, all polymers show similar π–π* absorption features characteristic of the anthracene-naphthalene backbone, indicating that the different alkyl/alkoxy groups only weakly perturb the conjugated core electronically. However, upon aggregation, distinct differences emerge. Polymers with shorter and less bulky substituents display a relatively modest enhancement in fluorescence intensity, whereas those carrying longer or more sterically demanding side chains exhibit a more pronounced AIE effect and stronger emission in the aggregated state. We ascribe this behavior to the increased ability of the bulkier side chains to restrict intramolecular motions and to modulate the packing of the PAH units, thereby suppressing nonradiative decay pathways. Overall, the optical data indicate that the naphthalene substituents mainly affect the aggregation process and AIE efficiency, rather than drastically altering the intrinsic electronic transitions of the multilayer backbone.

**Fig. 4:**
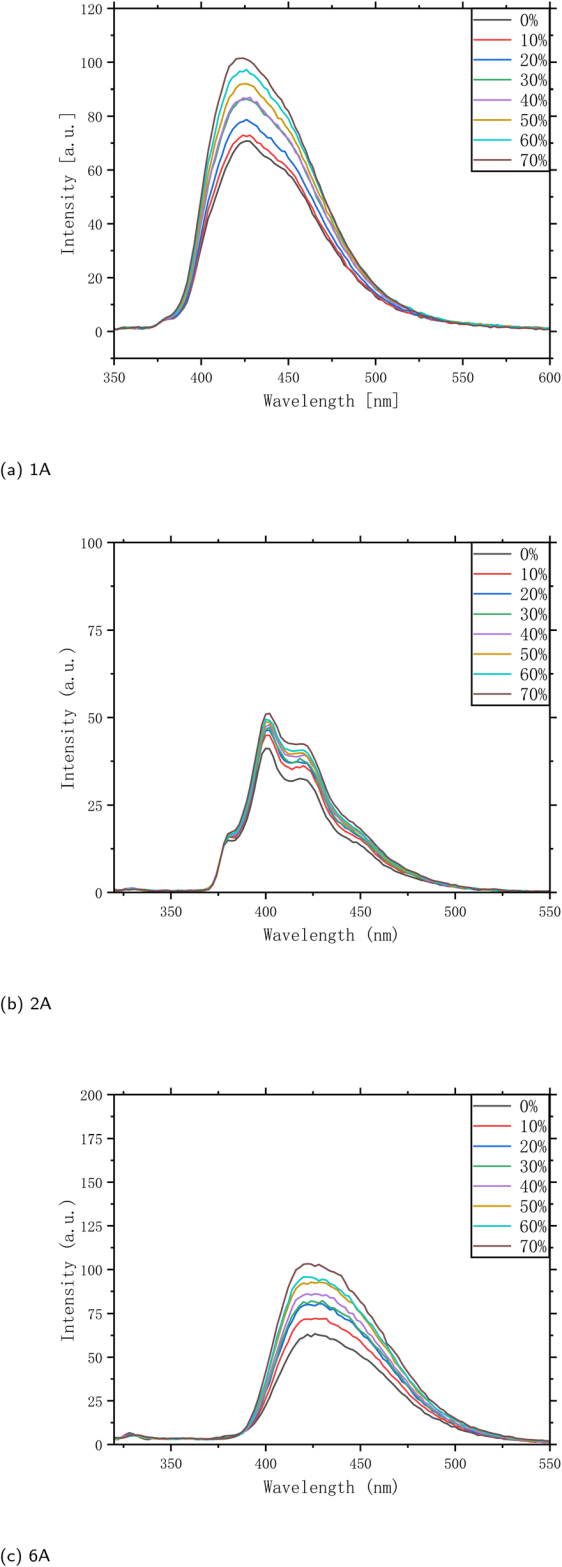
Photoluminescence (PL) spectra of polymers 1A, 2A, and 6A (*c* = 0.05 mg mL^−1^) recorded in THF/H_2_O mixtures at room temperature. The colored traces correspond to different volume percentages of water, *λ*_ex_ (1A) = 362 nm; *λ*_ex_ (2A) = 373 nm; *λ*_ex_ (6A) = 365 nm. Slit widths (*E*_*x*_/*E*_m_) = 5/5 nm. PMT high voltage: 600 V for 1A and 6A; 500 V for 2A.

Due to their differing substrates, polymers 1A, 2A, and 6A were selected for fluorescence studies in tetrahydrofuran (THF) at room temperature. As shown in the photoluminescence (PL) spectra (Fig. 4), the spectra of these polymers in THF at room temperature with varying water fractions (0–70% by volume). As the water content increases, the intensity of the emission increases steadily, reaching its maximum in the 400–430 nm range. This behavior exemplifies aggregation-induced emission (AIE), wherein the polymer is only weakly emissive in a good solvent (pure THF) but becomes strongly fluorescent upon aggregation.^24,25^ The addition of water reduces the solvent quality, promoting polymer aggregation and restricting intramolecular rotations and other non-radiative decay pathways. Consequently, the fluorescence is significantly enhanced in the aggregated state. Given its pronounced AIE properties, this naphthalene/anthracene-based polymer holds considerable promise as a highly sensitive platform for optical sensing and other optoelectronic applications that require robust fluorescence under aggregated conditions.^9,26^

**Fig. 5:**
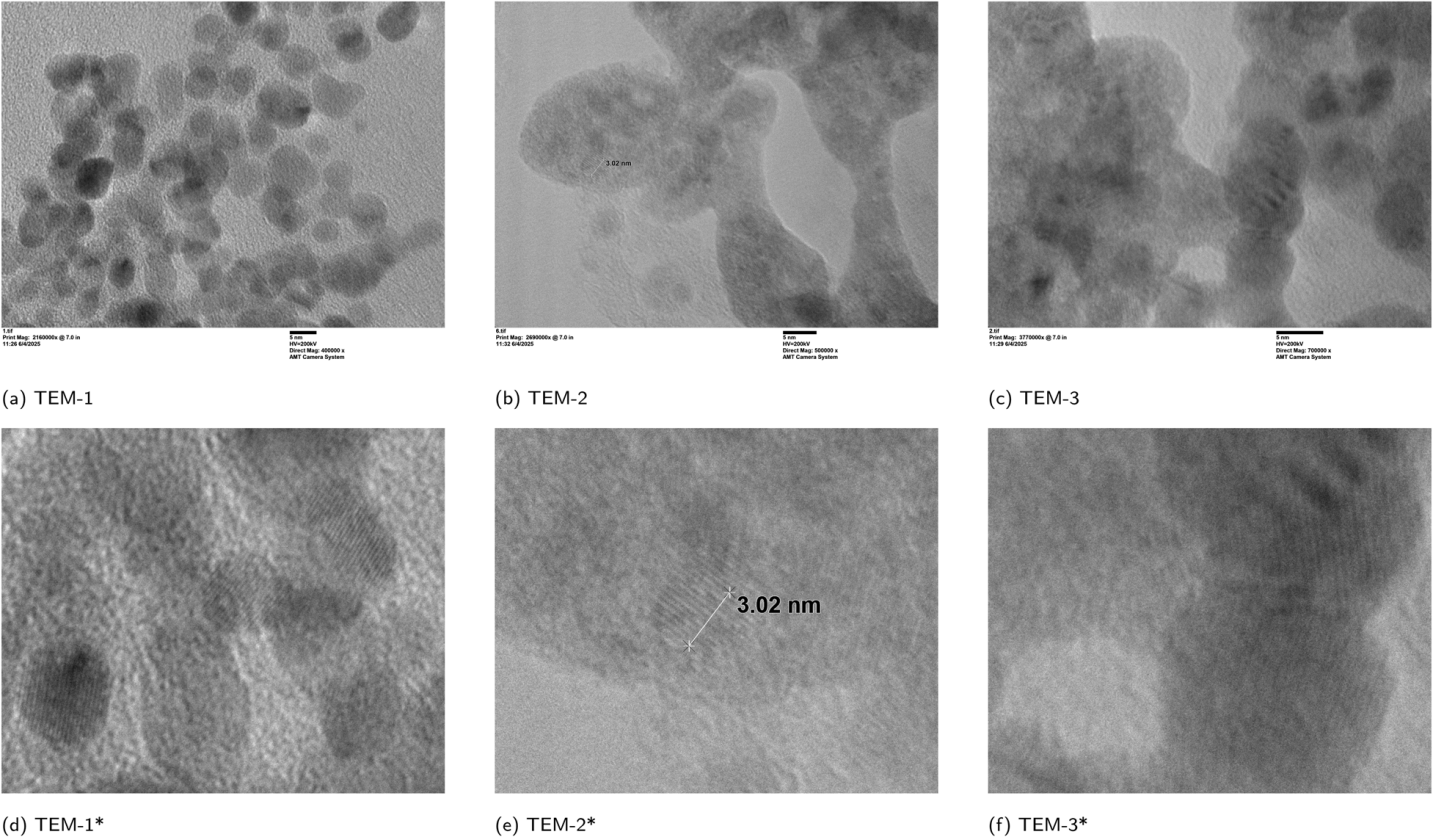
TEM image of polymer 1A: (a–c) representative TEM micrographs; (d–f) the corresponding locally magnified views of (a–c), respectively. Scale bar = 5 nm. Note: * indicates the locally magnified image.

The transmission electron microscopy (TEM) images of polymer 1A (Fig. 5) show heterogeneous contrast with nanoscale domains that are locally discernible in selected regions. Based on representative micrographs, the characteristic lateral dimensions of these domains are on the order of a few nanometres (approximately 3–6 nm). We note that TEM contrast, thickness variations, and projection effects can bias apparent domain boundaries; accordingly, we report these values as characteristic length scales rather than a rigorous size distribution.^27^

High-resolution regions display parallel lattice fringes; measuring across ∼10 periods yields an apparent periodicity of ∼0.30 nm per fringe. Considering the uncertainty associated with fringe assignment and projection/orientation effects, this value is comparable in order of magnitude to interchain π–π stacking distances commonly reported for semicrystalline conjugated polymers (typically ∼3.5–3.7 Å by GIWAXS, depending on chemistry and processing), and direct HRTEM visualization of ∼3.6 Å lattice spacing in conjugated polymers has been reported.^28–30^ Because lattice fringes are observed only when local ordered regions satisfy the imaging condition, nearby regions without visible fringes may reflect different orientations rather than being fully amorphous.^27^ The persistence of fringes over ∼10 periods thus suggests short-range periodic order in selected regions along the stacking direction, rather than long-range lamellar order throughout the sample.

In parallel, DFT calculations (Fig. 9) indicate a transition toward more uniform π-electron delocalization as the layer number increases, suggesting that monomer sequence and layer count can tune the frontier-orbital distribution and the electronic gap across 1A–6A.^31–33^ Overall, the TEM images support the presence of locally ordered nanoscale domains in polymer 1A, with periodic contrast consistent with short-range interchain ordering.

**Fig. 6:**
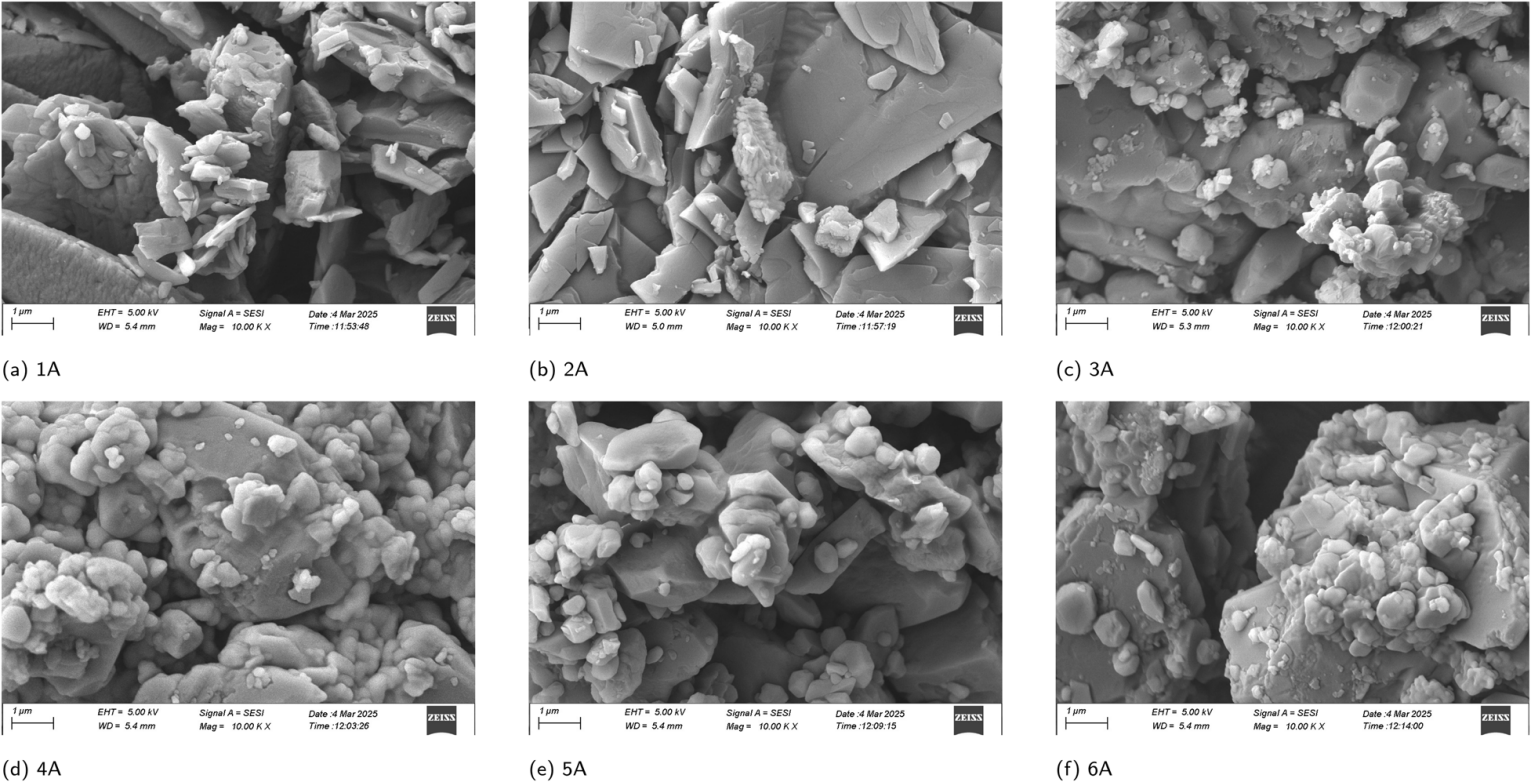
The Scanning Electron Microscope (SEM) images of polymer 1A–6A, bar scale = 1 µm.

The scanning electron microscope (SEM) of polymers 1A–6A shows faceted plate- and block-like crystallites ranging from submicrometer to a few micrometers (Fig. 6). Smaller particles are often attached, and interparticle voids with step edges give the surface a rough, textured appearance. Across the series, close contacts and bridges between crystallites suggest good mesoscale connectivity in the solid. Taken together with the TEM-derived π–π stacking metric (Fig. 5), the nano to micro organization supports an interconnected three-dimensional framework linked to improved charge transport and device performance in conjugated-polymer films.^31,34,35^

**Fig. 7:**
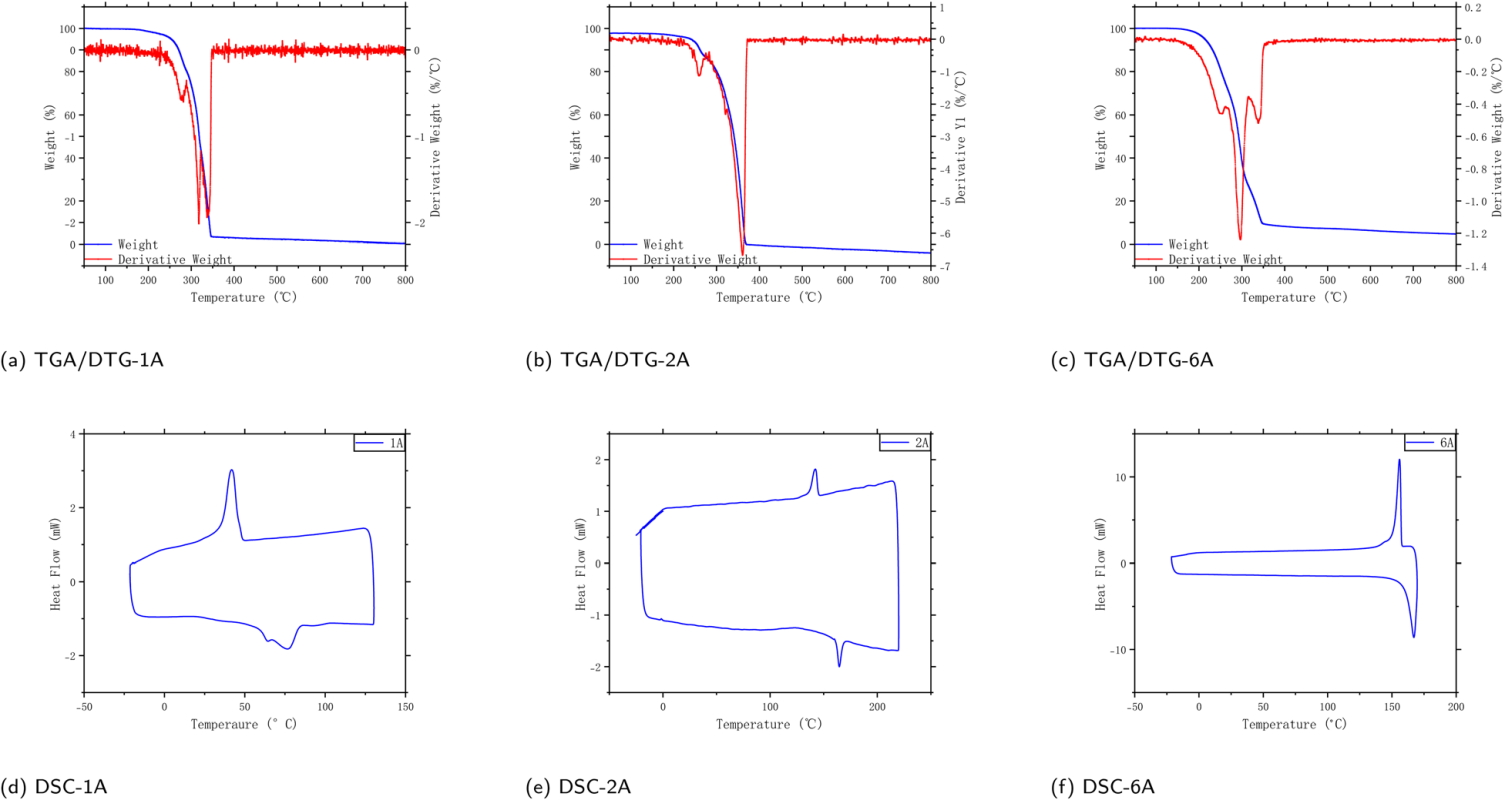
Thermogravimetric analysis (TGA), derivative thermogravimetric (DTG) and differential scanning calorimetry (DSC) analysis of polymers 1A, 2A, and 6A in N_2_ at a controlled heating rate. In DTG figure: left *Y*: weight remaining (%). Right *Y*: DTG = −d(mass%)/d*T* in %·°C^−1^. The DTG peak temperature *T*_max_ marks the principal degradation step; *T*_5%_, *T*_10%_ and the final residue are reported according to ISO 11358-1.^36^

The thermal properties and molecular characteristics of the synthesized polymers 1A, 2A, and 6A were investigated using thermogravimetric analysis (TGA) and differential scanning calorimetry (DSC) under nitrogen at a controlled heating rate.^37–39^ In the TGA/DTG profiles of these polymers (Fig. 7), a minor early mass loss is observed with *T*_5%_ in the 250–300 °C range, which is consistent with volatilization/desorption of residual low-molar-mass species rather than backbone scission. Following standard practice, the first-derivative curve (DTG = −d(mass%)/d*T*, %·°C^−1^) is used to identify the principal decomposition step *via* the DTG peak temperature *T*_max_.^36^ The single dominant degradation event is captured *T*_max_ at higher temperatures (Table 3), followed by a negligible residue above ∼450 °C in N_2_. Reporting *T*_5%_, *T*_10%_, *T*_max_, and residue lets us compare thermal stability across samples in a standard way, as recommended in ISO 11358-1.^36^ Such single–step decomposition with low residue strongly indicates chemically uniform, high-purity polymers with minimal volatile impurities. This behavior is consistent with known thermal profiles of aromatic polymers functionalized with anthracene, in which robust polycyclic cores contribute to elevated decomposition temperatures.^40,41^

**Table 3:** Summary of TGA/DTG metrics under N_2_ (programmed heating rate)^a^

Sample	*T*_5%_ (°C)	*T*_10%_ (°C)	*T*_max_ (°C)	Final residue (%)
1A	250.0	310.0	340.0	∼0
2A	255.0	270.0	360.0	0.10
6A	215.0	235.0	300.0	0.41

a Residue values are read at the high-*T* end of the run; small negative offsets (if any) are attributed to buoyancy/baseline drift.

Thermogravimetric and calorimetric measurements also show a consistent, if subtle, dependence on substituent type. All polymers exhibit high thermal stability with decomposition temperatures well above the typical operating range of organic optoelectronic devices, reflecting the inherent robustness of the anthracene and naphthalene cores. Within this overall picture, polymers with bulkier or longer side chains tend to display slightly lower onset decomposition temperatures and somewhat broader thermal transitions, which we attribute to their more flexible peripheral environment and less-efficient packing in the solid state. By contrast, polymers derived from monomers with more compact substituents exhibit marginally sharper thermal events, consistent with a higher degree of packing efficiency. These trends underscore that the naphthalene substituents, while not compromising the thermal robustness of the multilayer framework, do modulate the detailed thermal response through their impact on packing and segmental mobility.

The good thermal stability (onset metrics around ∼300–350 °C), combined with clean decomposition and consistent structural integrity, enables mid-temperature uses where thermal robustness, structural purity, and predictable degradation behavior are desirable—for example, in electronics, coatings, and thermally demanding barrier/insulation settings.

Differential scanning calorimetry (DSC) was used to further probe the thermal transitions of polymers 1A, 2A, and 6A (Fig. 7). The thermograms exhibit an exothermic event assignable to crystallization together with a subsequent endothermic melting peak on heating, a sequence commonly observed for semicrystalline polymer systems. These transitions provide a calorimetric signature of reversible ordering/disordering processes rather than direct proof of a single, uniform morphology.^42^

DSC peak temperatures and peak shapes can shift with heating rate and with the sample's prior thermal history.^43^ In addition, broadened or multiple melting features are often discussed as reflecting overlapping melting and reorganization processes during the scan, such as lamellar-thickness heterogeneity and/or melt-recrystallization.^43^ We therefore interpret the DSC traces conservatively as evidence that at least a fraction of the material shows thermally driven ordering that is lost upon heating. For 1A, this interpretation is qualitatively consistent with the locally ordered regions observed by TEM; however, DSC alone cannot uniquely determine lamellar periodicity or domain topology.

**Fig. 8:**
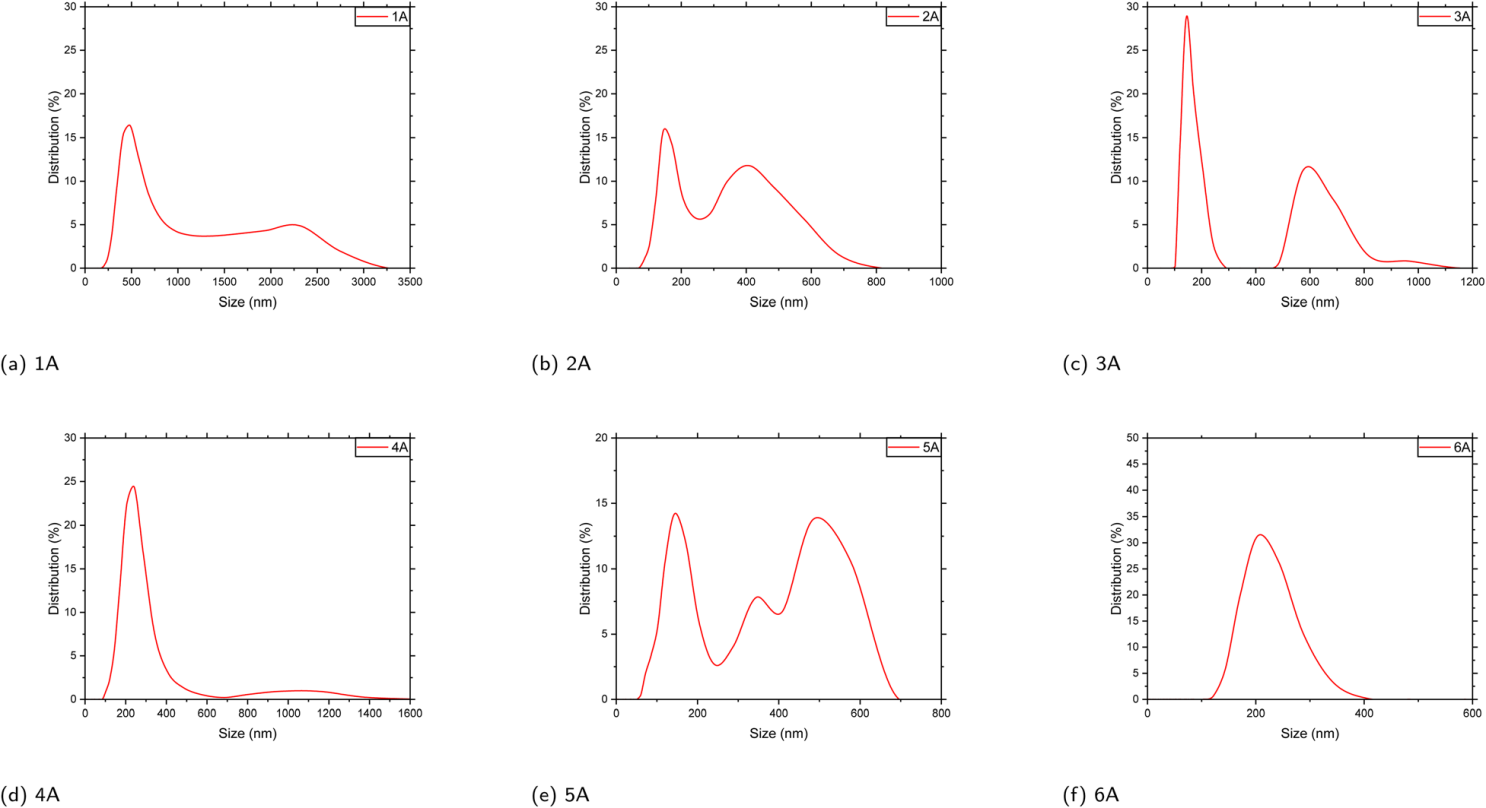
The Dynamic Light Scattering (DLS) of the polymer product 1A–6A, *c* = 0.4 mg mL^−1^ in MeOH/THF (50/50).

Dynamic light scattering (DLS) measurements of polymers 1A–6A (Fig. 8) show sample-specific, intensity-weighted apparent hydrodynamic size distributions in the submicrometer regime. Polymer 1A exhibits a broad distribution centered at ∼486 nm. Polymer 2A is bimodal with peaks at ∼172 nm and ∼344 nm. Polymer 3A is bimodal with peaks at ∼172 nm and ∼590 nm. Polymer 4A is close to a single mode centered at ∼243 nm. Polymer 5A is bimodal with peaks at ∼145 nm and ∼509 nm, with broadening into the mid-hundreds of nanometers. Polymer 6A is close to a single mode centered at ∼243 nm. Overall, the series is dominated by submicrometer populations; 2A/3A/5A retain stable secondary peaks, whereas 4A and 6A are closer to a single mode, and 1A shows the broadest distribution.

As a solution-state technique, DLS reports the hydrodynamic size of scattering objects in dispersion and therefore reflects the soluble/dispersible fraction of the polymer; in addition, the intensity-weighted representation is more sensitive to larger scatterers. The observed submicrometer populations and bimodality are in line with hierarchical aggregation behavior commonly discussed for conjugated polymers in solution.^44^

**Fig. 9:**
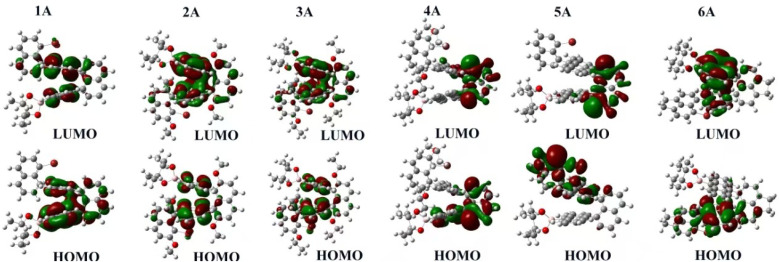
The ground-state density functional theory (DFT) calculations of polymers 1A–6A.

To investigate the electronic structure of anthracene-naphthalene polymers (1A to 6A), we conducted ground-state DFT calculations and present the HOMO/LUMO isosurfaces in Fig. 9.^45^ For polymers 1A–3A, the HOMO remains centered on the anthracene core with only slight extension into the flanking naphthalenes, while the LUMO is similarly confined to the peripheral rings. In 5A, the HOMO is almost exclusively localized on the anthracene unit, whereas its LUMO is restricted to one naphthalene flank, indicating asymmetric delocalization. In contrast, 6A exhibits both HOMO and LUMO fully delocalized across the central anthracene and both naphthalene side units. This progression reveals that once four or more anthracene-naphthalene layers are present, the system undergoes a transition to uniform π-delocalization, confirming that the monomer sequence and number of layers precisely regulate the orbital distribution and energy separation in these conjugated polymers.

## 3 Conclusion

In this study, we synthesized a series of conjugated multilayer 3D polymers (1A to 6A) from anthracene and naphthalene monomeric units *via* Suzuki–Miyaura polycoupling. These polymers show strong π–π* absorption in the UV range and pronounced aggregation-induced emission (AIE) in the aggregated state, supporting their potential relevance to UV-blocking coatings, photonic materials, optical sensing, and bioimaging. Thermal analyses indicate good stability, with decomposition occurring above 250 °C and well-defined thermal transitions, consistent with thermally robust aromatic polymer frameworks. Electron microscopy provides complementary structural insight: SEM suggests layered surface textures without obvious macroscopic phase separation, while TEM images reveal nanoscale domains with locally ordered contrast features. In selected high-resolution regions, parallel fringes can be observed with apparent spacings on the order of 3.0–3.5 Å, consistent with short-range stacking order within these domains.^46^ Taken together, the microscopy results support locally ordered packing motifs in the solid state and provide a structural context for the aggregation-related optical behavior discussed above. In solution, dynamic light scattering (DLS) yields predominantly submicrometer, intensity-weighted apparent hydrodynamic size distributions for the soluble/dispersible fraction: 4A and 6A tend toward single-mode profiles, whereas 1A, 2A, 3A, and 5A show broader or bimodal profiles. Given the intrinsic intensity weighting of DLS, these distributions are consistent with aggregate-rich dispersions commonly observed for conjugated polymers in solution. Complementary DFT calculations based on oligomer models map frontier-orbital distributions and indicate a layer-dependent increase in π-delocalization as the layer number increases. Taken together, these results establish the 1A–6A series as a useful platform for exploring structure–photophysics relationships in multilayer PAH-based polymers and for guiding future development of functional aromatic materials.

## Author contributions

H. L. carried out the synthesis, characterization, and sensing experiments. G. L. supervised the project, designed the experiments, and revised the manuscript. Both authors reviewed and approved the final manuscript.

## Conflicts of interest

There are no conflicts to declare.

## Supplementary Material

RA-016-D5RA08369A-s001

## Data Availability

All data supporting the findings of this study—including experimental procedures, characterization datasets and additional analyses—are available in the supplementary information (SI). Supplementary information is available. See DOI: https://doi.org/10.1039/d5ra08369a.
