# Supplementary material for: Design, synthesis, and analysis of multi-layered 3D fluorescent polymers derived from anthracene and naphthalene structural units
Source: RSC Adv. 2026 Jan 15;16(4):3420–9. doi: 10.1039/d5ra08369a (PMC12805467; doi:10.1039/d5ra08369a)
Supplement: RA-016-D5RA08369A-s001 [file RA-016-D5RA08369A-s001.pdf]

Supplementary Information (SI) for RSC Advances.
This journal is © The Royal Society of Chemistry 2026

Supporting Information

Design, Synthesis, and Analysis of Multi-Layered 3D Fluorescent Polymers Derived from Anthracene and Naphthalene Structural Units

Table of Contents

1. **General Information** 3
2. **Synthetic Procedures** 4
   2.1. **Polymerization Procedure** 6
3. **NMR Spectrum** 8
4. **GPC Spectrum of polymers** 17

References 23

1. General Information

Unless otherwise stated, all reactions were magnetically stirred and conducted in oven-dried glassware in anhydrous solvents under Ar. Solvents and liquid reagents, as well as solutions of solid or liquid reagents were added via syringes, stainless steel or polyethylene cannulas through rubber septa or through a weak Ar counter-flow. Cooling baths were prepared in Dewar vessels, filled with ice/water (0 °C) or dry ice/acetone (-78 °C). Heated oil baths were used for reactions requiring elevated temperatures. Solvents were removed under reduced pressure at 40-65 °C using a rotavapor. All given yields are isolated yields of chromatographically and NMR spectroscopically materials.

All abbreviations used in the Article and in this Supporting Information are defined below, ethyl acetate (EA, EtOAc), diethyl ether (ether, Et_2O), dichloromethane (DCM), chloroform ($CHCl_3$), tetrahydrofuran (THF), 1,4-dioxane (dioxane), dimethylformamide (DMF), dimethyl sulfoxide (DMSO), N-methyl-2-pyrrolidone (NMP), acetonitrile (MeCN, ACN), methanol (MeOH), ethanol (EtOH), isopropyl alcohol (IPA, i-PrOH), n-butanol (n-BuOH), tert-butanol (t-BuOH), acetone (acetone), toluene (PhMe), hexane (Hex), pyridine (Py), water (H_2O).

All commercially available chemicals were used as received without further purification. Solvents as follows: CH_3OH, toluene, EA, ether, DCM, dioxane, acetone were used without further purification. THF and DCM are delivered from an Innovation Technology solvent system.

The ^1H and ^{13}C NMR spectra were recorded in chloroform-*d*, DMSO-d_6 and THF-d_8 on 400 MHz and 500 MHz instruments with TMS as internal standard. For referencing the ^1H NMR spectra, the residual solvent signal (δ = 7.26 for chloroform-*d*, δ = 2.50 for DMSO-d_6 and δ = 1.85 and 3.76 for THF-d_8) were used. In the case of the ^{13}C NMR spectra, the signal of solvents (δ = 77.16 for chloroform-*d*, δ = 39.52 for DMSO-d_6 and δ = 25.62 and 67.97 for THF-d_8) were used. Chemical shifts(δ) were reported in ppm with respect to TMS. Data are represented as follows: chemical shift, multiplicity (s = singlet, d = doublet, t = triplet, m = multiplet), coupling constant (J, Hz), and integration. Samples 1A and 6A were characterized by DLS (Nanotrac NPA250) in MeOH/THF (50/50), the sample concentration is 0.4 mg/mL. Samples 1A and 6A were canned by SEM Zeiss crossbeam 540, at 3 kV accelerating voltage with secondary electron detector. TEM Hitachi 8100, 200 Kv accelerating voltage. Samples solutions in EtOH were dropped on TEM grid substrates and were then inspected by TEM (H8100) at an accelerating voltage of 200 Kv. GPC data were collected using TOSOH Eco SEC HLC-8320 GPC equipped with a dual-flow refractive index detector. A UV detector is also included for UV visible polymers and can be used in tandem with the RI detector. The installed columns have a range of 500 – 107 Da. Samples were run for 20 minutes with flow rate 0.7 mL/min. Polystyrene (PS) standards (PstQuick C) were used for calibration in our experiments. The NMR data were collected using JEOL ECS 400 MHz NMR Spectrometer with multinuclear, direct detection probe, automatic sample changer, variable temperature, and Z-gradient capabilities. High Resolution mass spectrometer Orbitrap Fusion

Lumos from Thermo Scientific, Palo Alto, CA was used at resolution of 120000 using ESI and infusion using acetonitrile as solvent and 1 microgram per milliliter of sample concentration.

2. Synthetic Procedures

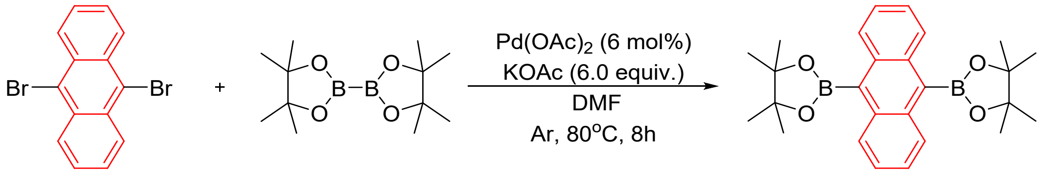

9,10-bis(4,4,5,5-tetramethyl-1,3,2-dioxaborolan-2-yl) anthracene (**A**)

9,10-dibromoanthracene (5.40 g, 16 mmol), KOAc (9.80 g, 39.0 mmol), Palladium (II) acetate (215.52 mg, 0.96 mmol) and 100 mL of Dimethylformamide were introduced into a 250 mL round-bottom flask equipped with a stirring bar and a rubber septum. The flask was then degassed under vacuum and backfilled with argon three times. The mixture was heated up to 80 °C, 8 hours. After the reaction is completed, Cool the mixture to room temperature and pour the mixture into water while stirring. Extract the mixture with dichloromethane, then wash the organic layer with brine. Dry the organic layer over anhydrous sodium sulfate. After rotary evaporation, recrystallize the residue from EtOH.[1] The resulting yellow solid. ^{1}H NMR (400 MHz, chloroform-*d*) δ 8.37 – 8.28 (m, 4H), 7.47 – 7.41 (m, 4H), 1.57 (s, 24H). ^{13}C NMR (101 MHz, chloroform-*d*) δ 135.01, 128.89, 125.27, 84.59, 25.30. HRMS (ESI) m/z: $[M + H]^+$ Calcd for $\mathbf{C_{26}H_{33}B_2O_4}$ 431.2559; Found 431.2560

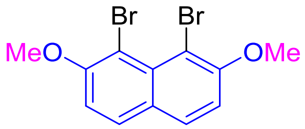

2

2 was synthesized from 2,7-dihydroxynaphthalene following the reported procedure.[2-5] **1,8-dibromo-2,7-dimethoxynaphthalene (2):** Yellow solid, 76%. ^{1}H NMR (400 MHz, chloroform-d) δ 7.66 (d, J = 8.9 Hz, 2H), 7.08 (d, J = 8.9 Hz, 2H), 3.94 (s, 6H). ^{13}C NMR (101 MHz, chloroform-*d*) δ 156.56, 131.76, 130.19, 127.50, 111.75, 106.09, 57.30. HRMS (ESI) m/z: $[M+H]^+$ (M+2 isotope peak) Calcd for $\mathbf{C_{12}H_{11}Br_2O_2}$ 346.9100; Found 346.9099.

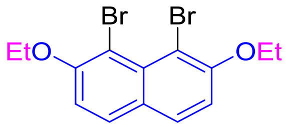

3

3 was synthesized from 2,7-dihydroxynaphthalene following the reported procedure. [2-5] **1,8-dibromo-2,7-diethoxynaphthalene (3):** Yellow solid, 71%. ^{1}H NMR (400 MHz, chloroform-*d*)

δ 7.62 (d, J = 9.0 Hz, 2H), 7.05 (d, J = 8.9 Hz, 2H), 4.18 (q, J = 7.0 Hz, 4H), 1.49 (t, J = 7.0 Hz, 6H). ^{13}C NMR (101 MHz, chloroform-*d*) δ 156.08, 132.33, 131.98, 129.98, 127.65, 127.57, 113.29, 106.97, 106.47, 66.15, 65.76, 15.14, 14.63. HRMS (ESI) m/z: $[M + H]^+$ Calcd for $\mathbf{C_{14}H_{15}Br_2O_2}$ 372.9433; Found 372.9440

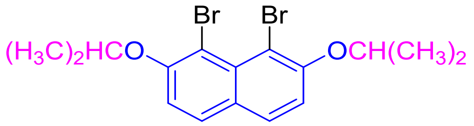

4

4 was synthesized from 2,7-dihydroxynaphthalene following the reported procedure. [2-5] **1,8-dibromo-2,7-diisopropoxy-naphthalene (4):** Yellow solid, 63%. ^{1}H NMR (400 MHz, chloroform-*d*) δ 7.69 – 7.62 (m, 2H), 7.11 (dd, J = 8.9, 1.7 Hz, 2H), 4.67 (m, J = 6.1, 1.7 Hz, 2H), 1.42 (dd, J = 6.1, 1.8 Hz, 12H). ^{13}C NMR (101 MHz, chloroform-*d*) δ 155.47, 132.48, 129.60, 128.00, 115.81, 109.13, 73.68, 29.79, 22.46, 22.03. HRMS (ESI) m/z: $[M + H]^+$ Calcd for $\mathbf{C_{16}H_{19}Br_2O_2}$ 400.9746; Found 400.9751

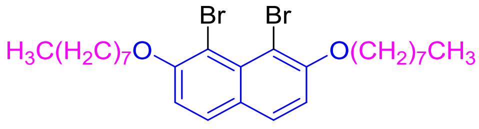

5

5 was synthesized from 2,7-dihydroxynaphthalene following the reported procedure. [2-5] **1,8-dibromo-2,7-bis(octyloxy)-naphthalene 5):** Yellow solid, 68%. ^{1}H NMR (400 MHz, chloroform-*d*) δ 7.62 (d, J = 8.9 Hz, 2H), 7.04 (d, J = 8.9 Hz, 2H), 4.07 (t, J = 6.5 Hz, 4H), 1.81 (dq, J = 8.5, 6.6 Hz, 4H), 1.50 – 1.39 (m, 4H), 1.36 – 1.13 (m, 16H), 0.86 – 0.78 (m, 6H). ^{13}C NMR (101 MHz, chloroform-*d*) δ 156.18, 131.98, 129.92, 127.44, 113.08, 106.84, 70.46, 31.91, 29.50, 29.40, 29.33, 26.15, 22.77, 14.22. HRMS (ESI) m/z: $[M]^+$ (M+2 isotope peak) Calcd for $\mathbf{C_{26}H_{38}Br_2O_2}$ 542.1218; Found 542.1217

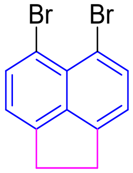

6

6 was synthesized from 2,7-dihydroxynaphthalene following the reported procedure. [2-5] **5,6-dibromo-1,2-dihydroacenaphthylene (6):** Yellow solid, 83%. ^{1}H NMR (400 MHz, chloroform-*d*) δ 7.79 (d, J = 7.4 Hz, 2H), 7.09 (dt, J = 7.5, 0.9 Hz, 2H), 3.30 (d, J = 0.9 Hz, 4H). ^{13}C NMR (101 MHz, chloroform-*d*) δ 147.19, 142.09, 135.96, 127.89, 121.06, 114.48, 30.15. HRMS (ESI) m/z: $[M + H]^+$ (M+2 isotope peak) Calcd for $\mathbf{C_{12}H_9Br_2O_2}$ 344.8943; found 344.8957.

2.1. Polymerization Procedure

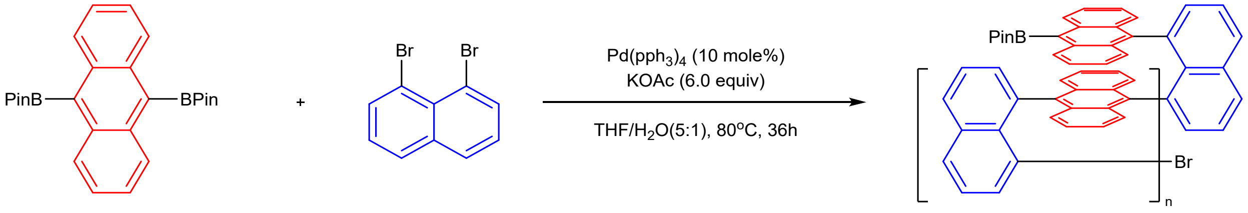

A

9,10-bis(4,4,5,5-tetramethyl-1,3,2-dioxaborolan-2-yl) anthracene (A)(172.06 mg, 0.4 mmol, 1 equiv), 1,8-dibromonaphthalene (114.4 mg, 0.4 mmol, 1.0 equiv), KOAc (235.54 mg, 2.4 mmol, 6.0 equiv) and $Pd(pph_3)_4$ (16 mg, 0.04 mmol) were added to a 15 mL oven-dried pressure vessel, There were 5 mL of THF and 1 mL of H_2O added to the pressure vessels' bottom. The pressure vessel was vacuum degassed before argon was introduced. Then, it was heated at 80 °C for 36 hours. The mixture was refrigerated and brought to room temperature. The resulting combination was then added to MeOH/HCl (5:1 %Vol) in a single pot. The precipitated components were filtered through a Buchner funnel, collected, and repeatedly washed with MeOH and water. Additional drying was done to create light yellow solid (266.40 mg, 93%) **(1A)**. M_n= 6681, M_w= 7642, PDI= 1.144. ^{1}H NMR (400 MHz, chloroform-*d*) δ 8.01 – 7.46 (m, Ar-H).

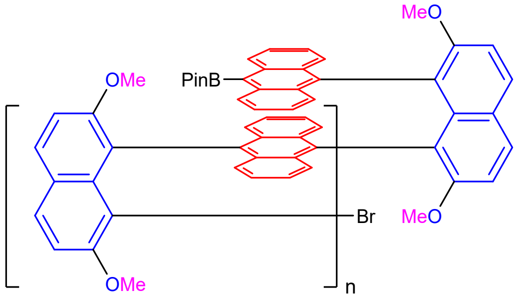

2A

The same synthesis procedure as Polymer **1A**, **2A** was obtained as light-yellow solid (158.2 mg, 51%) M_n=7006, M_w= 8050, PDI= 1.149. ^{1}H NMR (400 MHz, chloroform-*d*) δ 8.42 – 7.45 (m, Ar-H), 4.01 – 3.96 (dd, OCH_3-H).

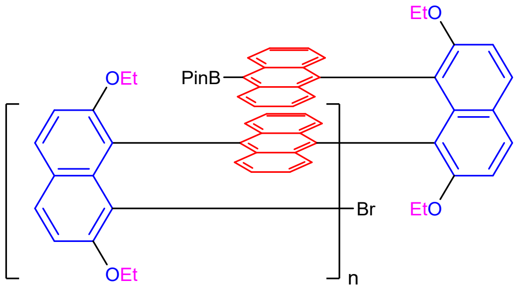

3A

The same synthesis procedure as Polymer **1A**, **3A** was obtained as white solid (173.3 mg, 54%). M_n= 5891, M_w= 6677, PDI=1.133. ^{1}H NMR (400 MHz, chloroform-*d*) δ 8.4 – 6.8 (m, Ar-H), 4.11 (m, OCH_2-H), 1.31 (s, CH_3-H).

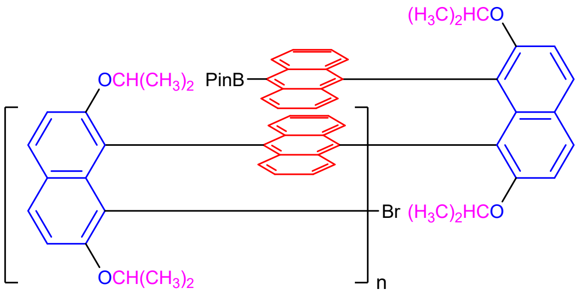

4A

The same synthesis procedure as Polymer **1A**, **4A** was obtained as white solid (169.7 mg, 51%). M_n=6533, M_w= 7639, PDI=1.169. ^{1}H NMR (400 MHz, chloroform-*d*) δ 8.36 – 7.07 (m, Ar-H), 4.6 - 4.8 (m, O*i*Pr-H), 1.50-1.42 (s, CH_3-H).

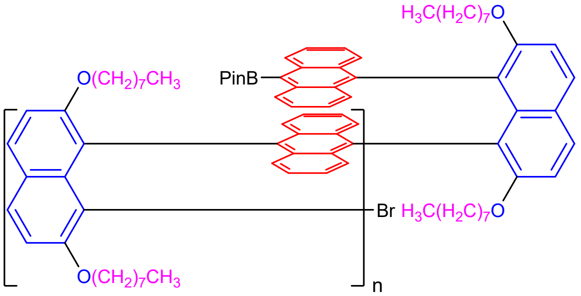

5A

The same synthesis procedure as Polymer **1A**, **5A** was obtained as white solid (331.1 mg, 85%). M_n= 6126, M_w=6668, PDI=1.088.^{1}HNMR (400 MHz, chloroform-*d*) δ 8.64 – 7.05 (m, Ar-H), 4.16 – 4.11 (m, OCH_2-H), 1.94 - 0.90 (m, C_8H_{17}-H).

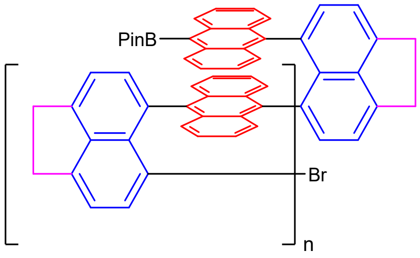

6A

The same synthesis procedure as Polymer **1A**, **6A** was obtained as white solid (178.12 mg, 86%). M_n= 7171, M_w= 7872, PDI= 1.098. ^{1}H NMR (400 MHz, chloroform-*d*) δ 8.36 – 7.06 (m, Ar-H), 3.30 – 3.27 (s, Ar-CH_2 CH_2-H).

3. NMR Spectrum

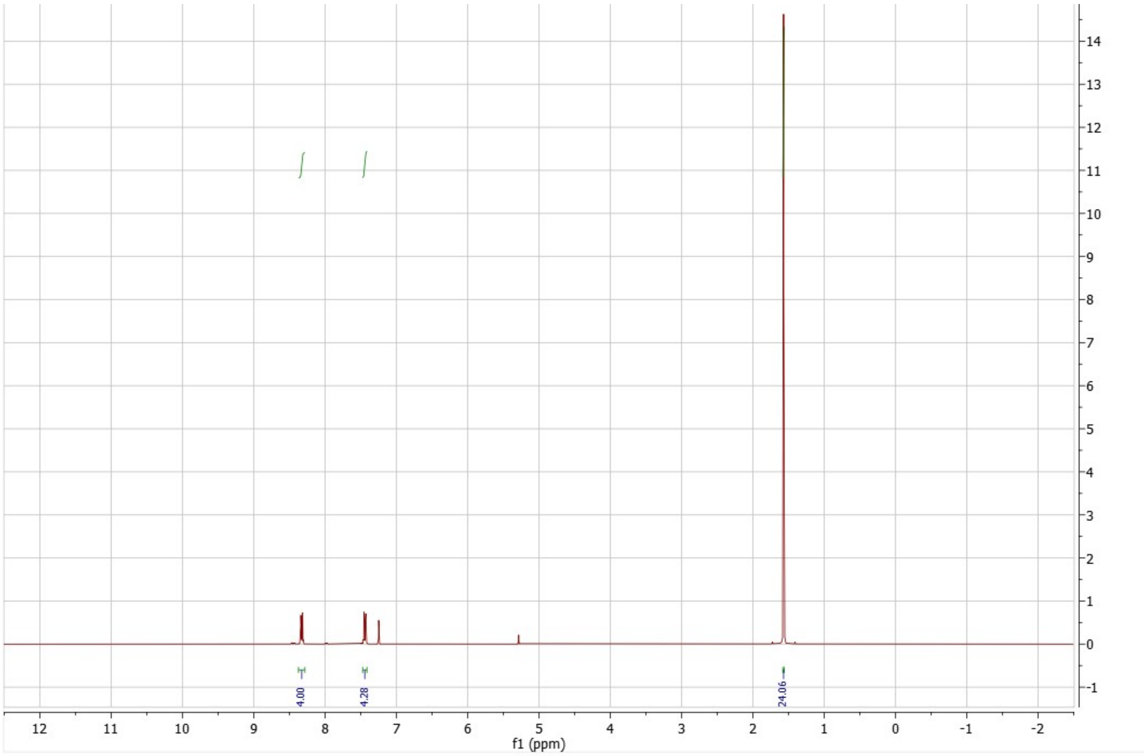

*Figure 1: ^{1}H NMR of 9,10-bis(4,4,5,5-tetramethyl-1,3,2-dioxaborolan-2-yl) anthracene (**A**)*

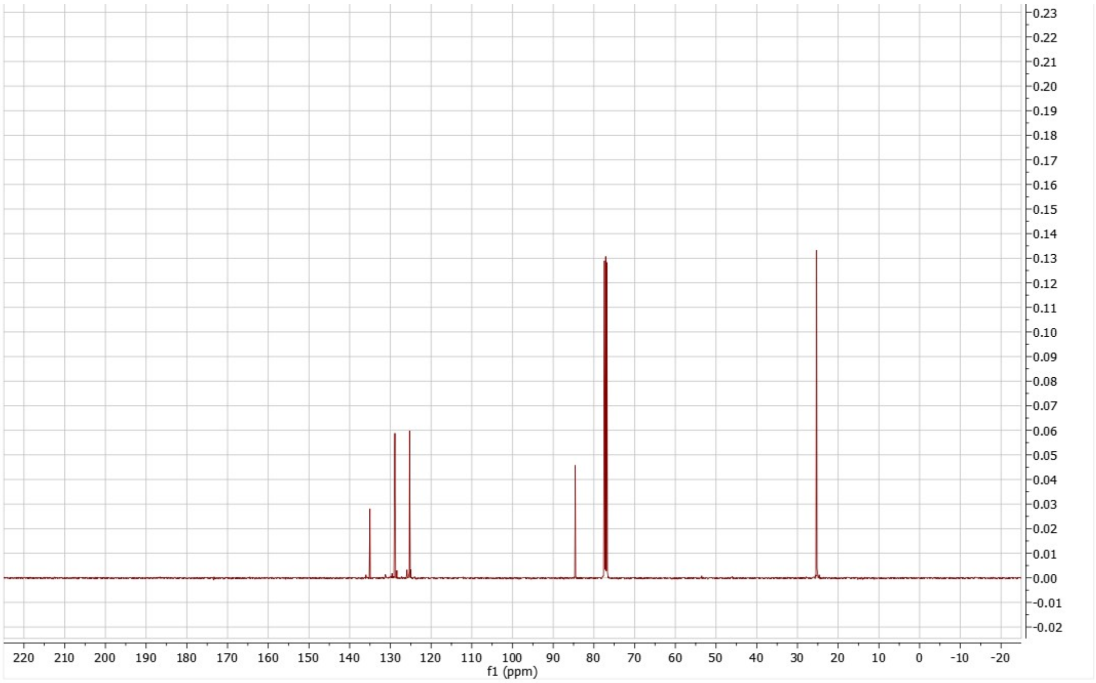

*Figure 2: ^{13}C NMR of 9,10-bis(4,4,5,5-tetramethyl-1,3,2-dioxaborolan-2-yl) anthracene (**A**)*

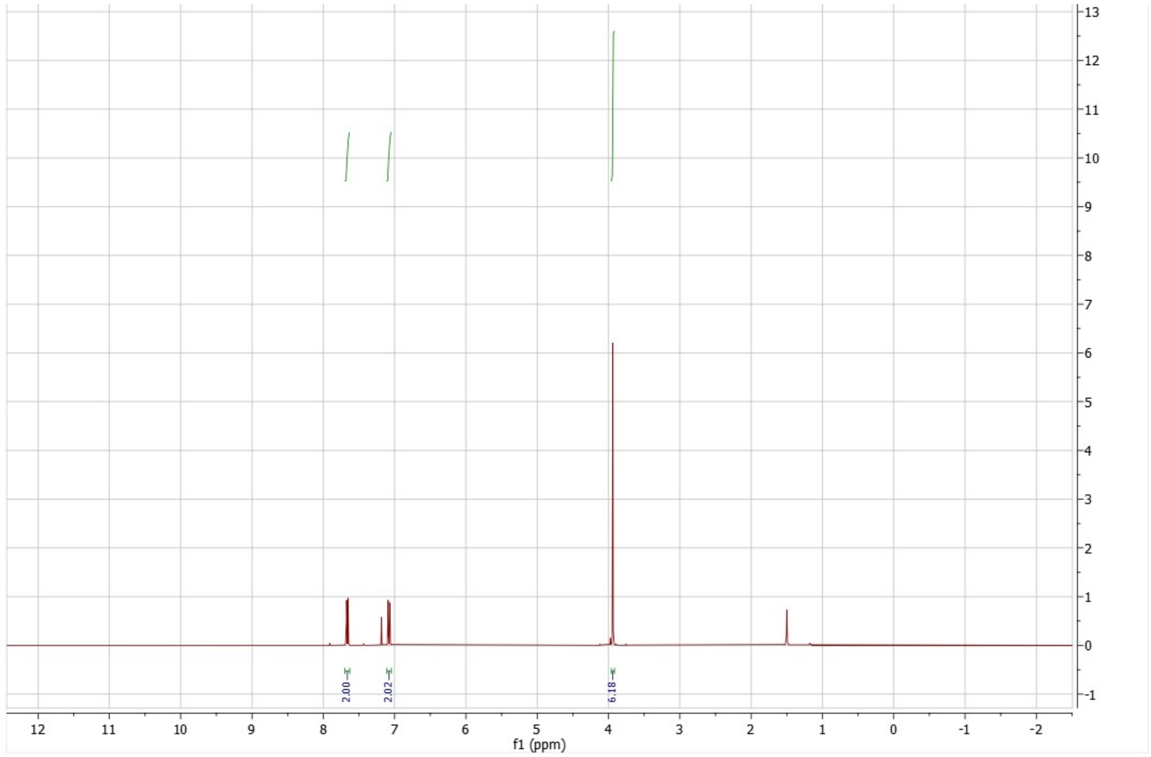

*Figure 3: ^{1}H NMR of 1,8-dibromo-2,7-dimethoxynaphthalene (**2**)*

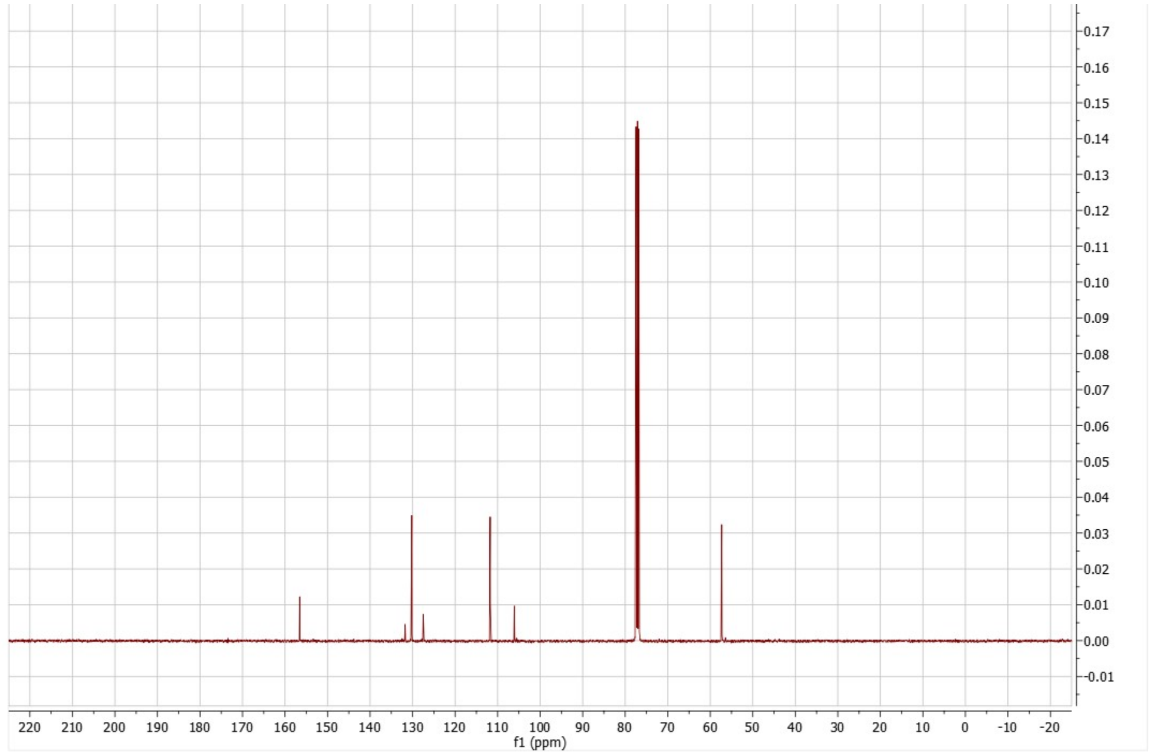

*Figure 4: ^{13}C NMR of 1,8-dibromo-2,7-dimethoxynaphthalene (**2**)*

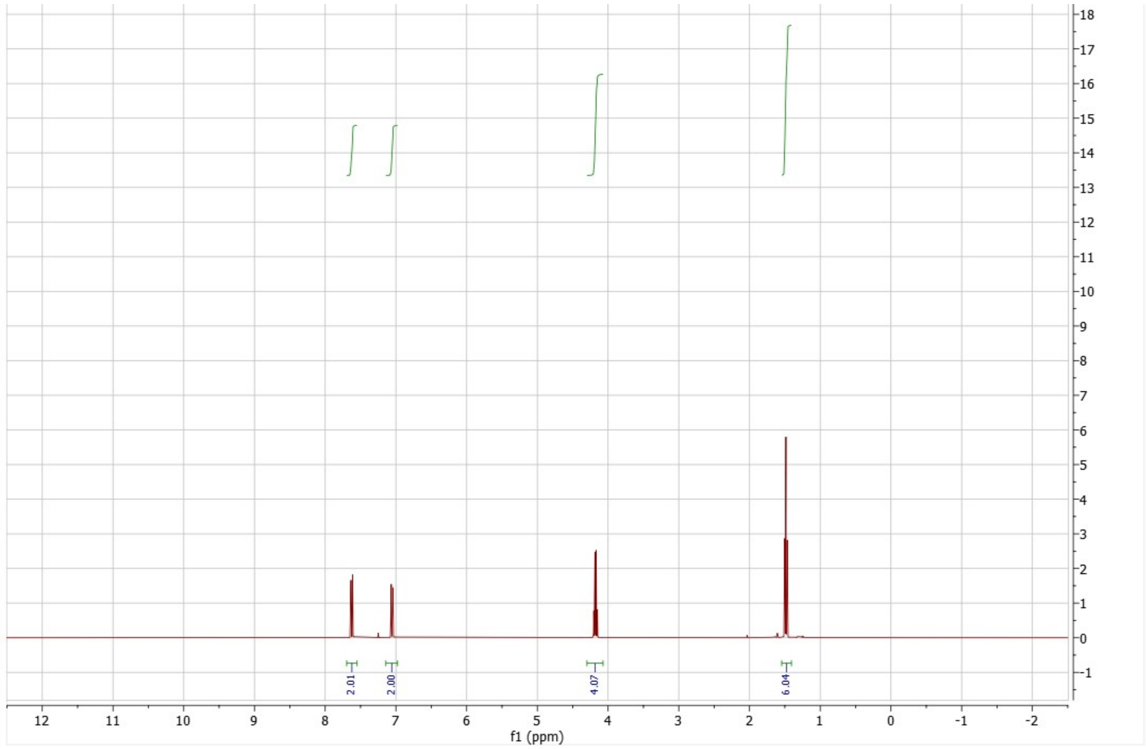

*Figure 5: ^{1}H NMR of 1,8-dibromo-2,7-diethoxynaphthalene (**3**)*

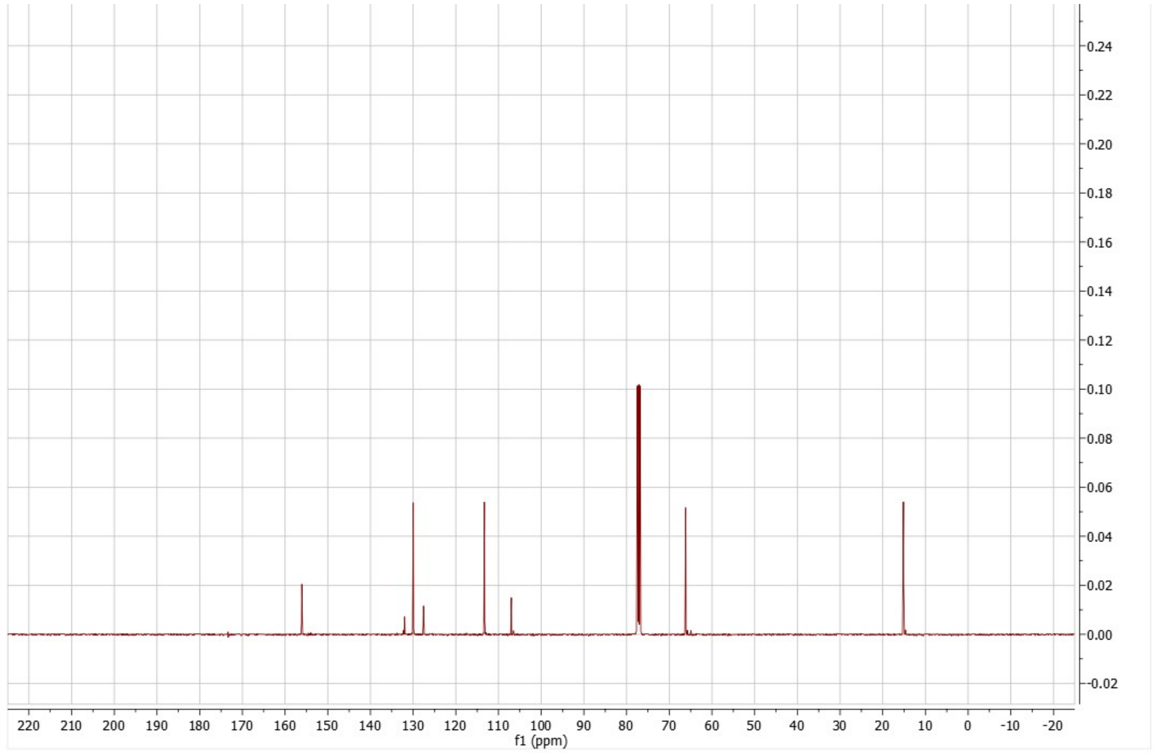

*Figure 6: ^{13}C NMR of 1,8-dibromo-2,7-diethoxynaphthalene (**3**)*

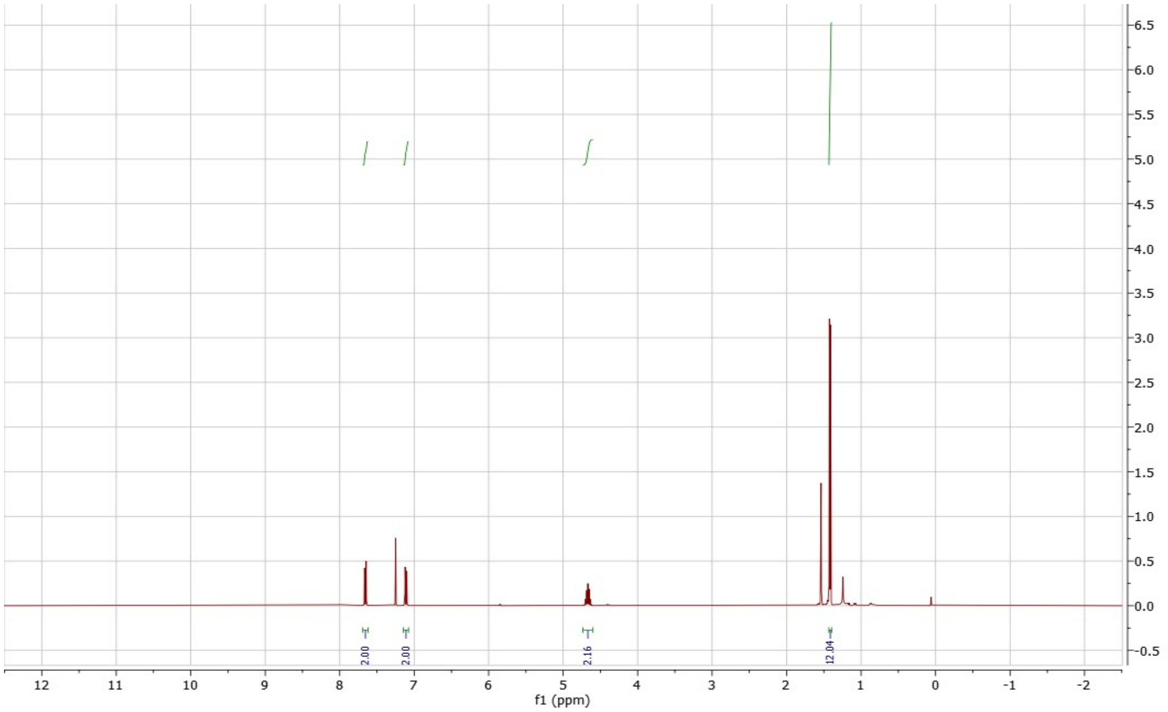

*Figure 7: ^{1}H NMR of 1,8-dibromo-2,7-diisopropoxynaphthalene (**4**)*

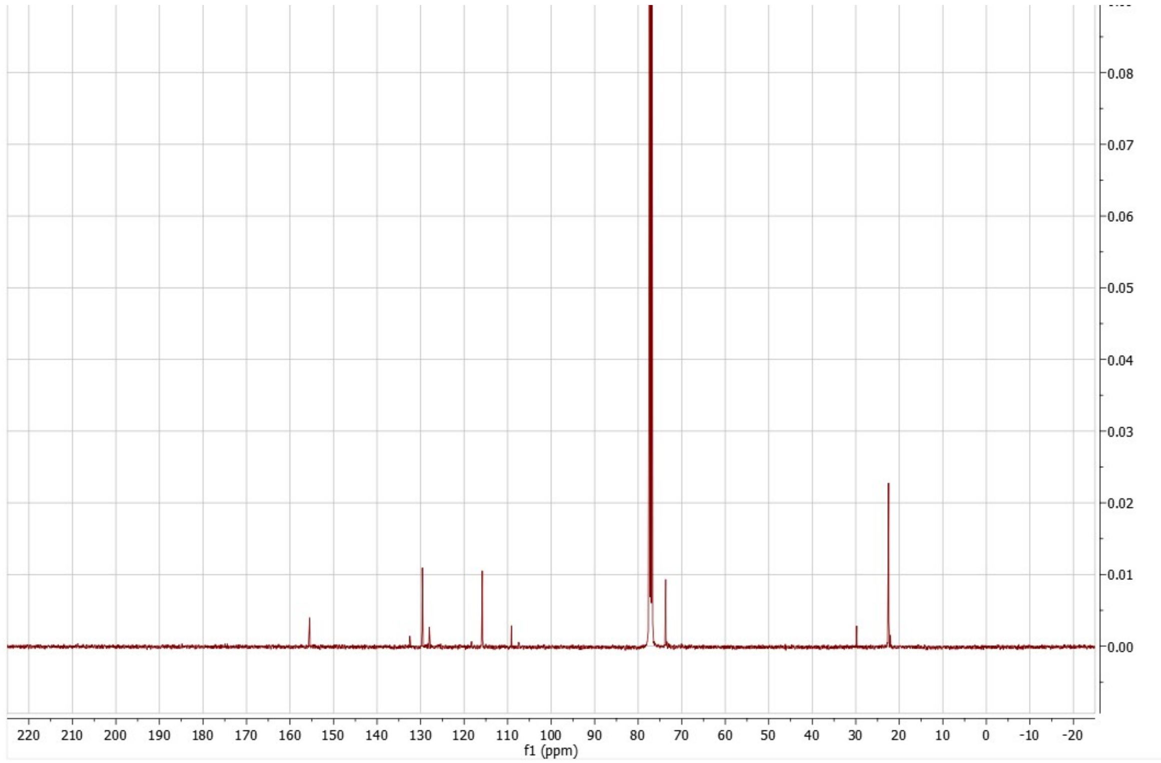

*Figure 8: ^{13}C NMR of 1,8-dibromo-2,7-diisopropoxynaphthalene (**4**)*

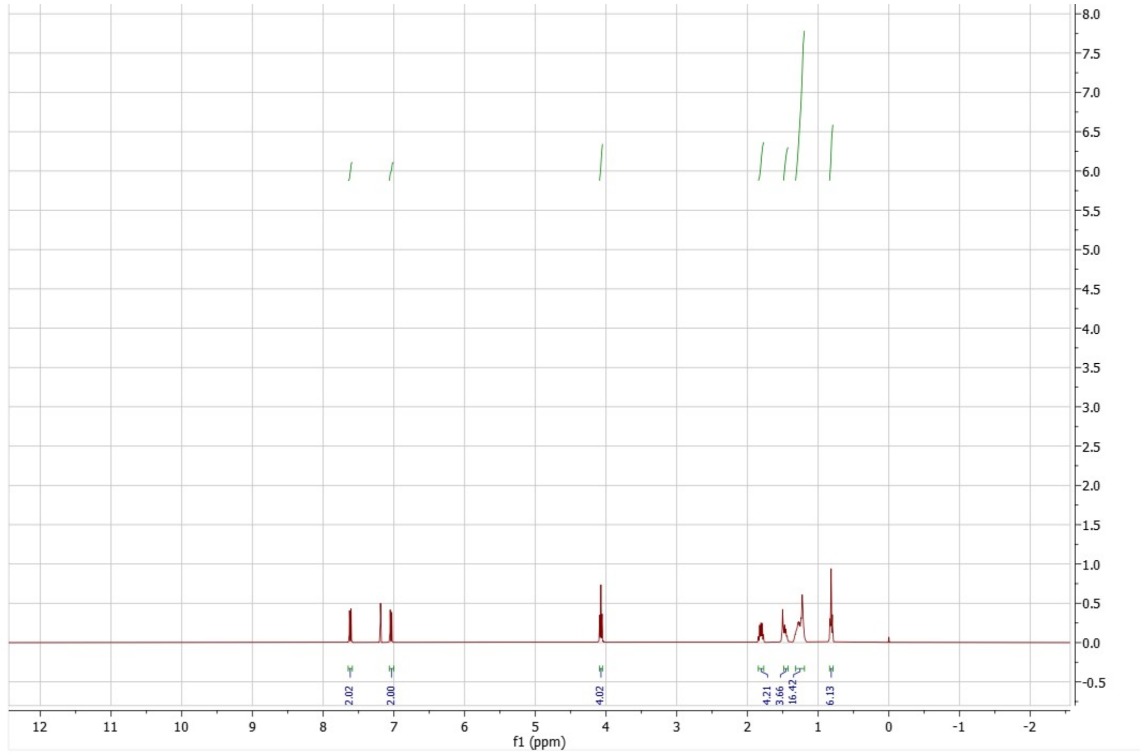

*Figure 9: ^{1}H NMR of 1,8-dibromo-2,7-bis(octyloxy)naphthalene (**5**)*

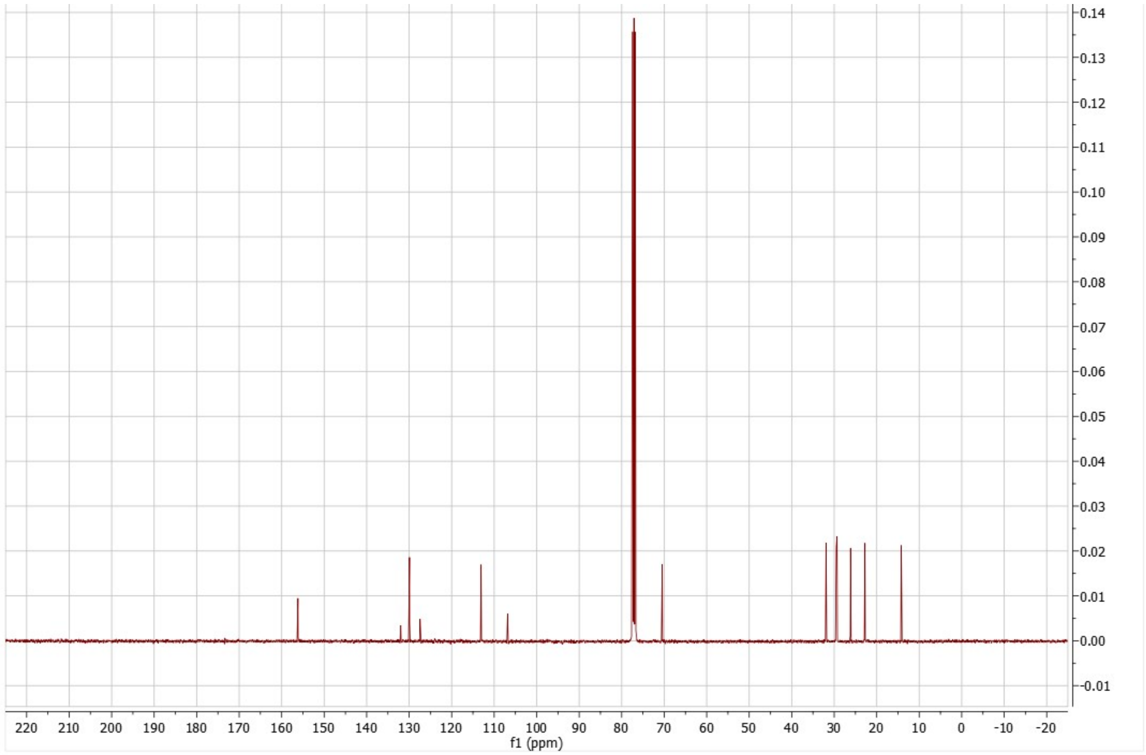

*Figure 10: ^{13}C NMR of 1,8-dibromo-2,7-bis(octyloxy)naphthalene (**5**)*

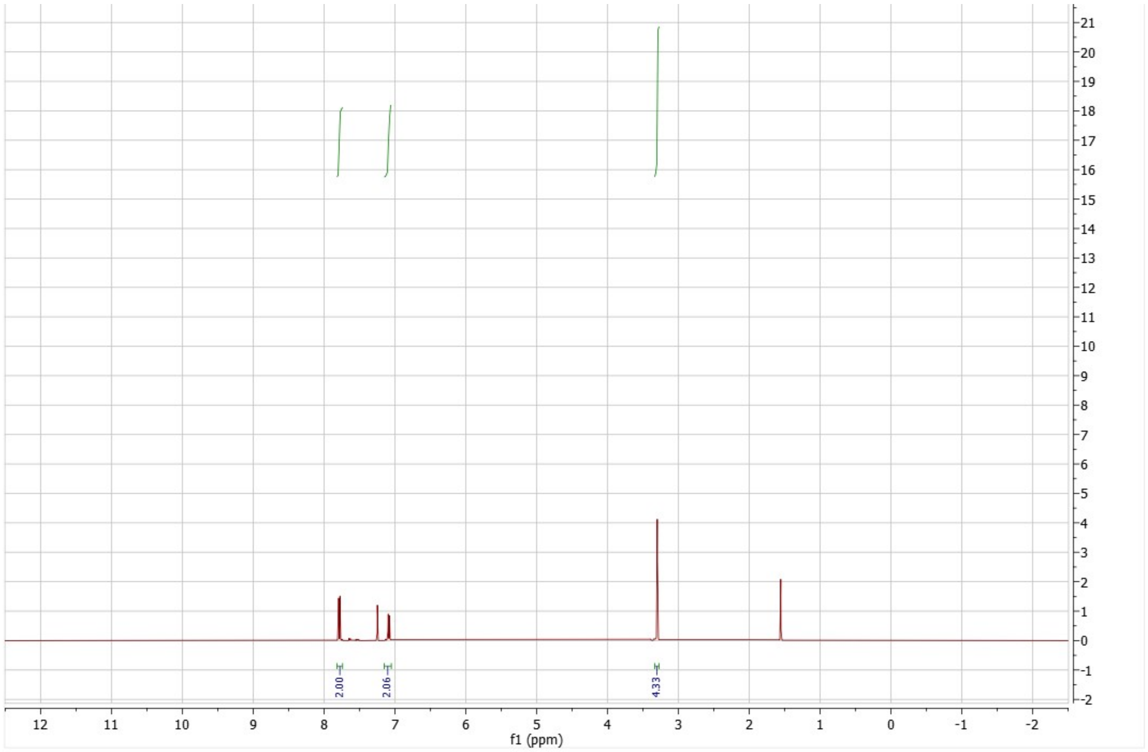

*Figure 11: ^{1}H NMR 5,6-dibromo-1,2-dihydroacenaphthylene (**6**)*

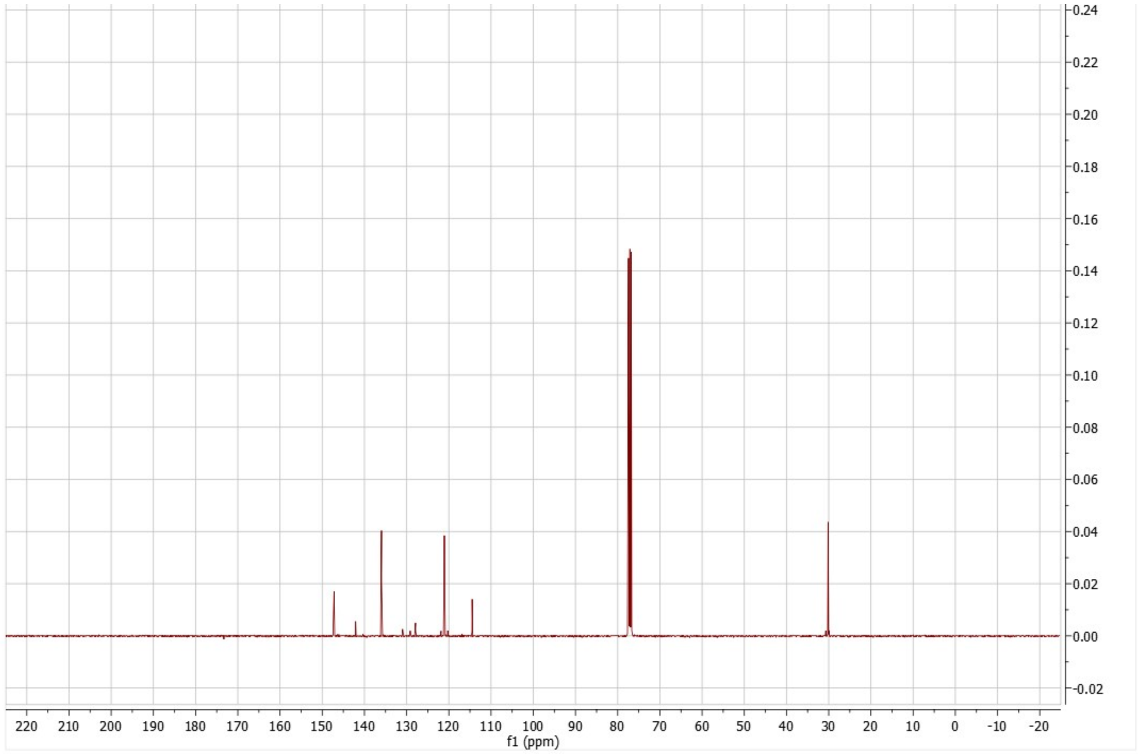

*Figure 12: ^{13}C NMR 5,6-dibromo-1,2-dihydroacenaphthylene (**6**)*

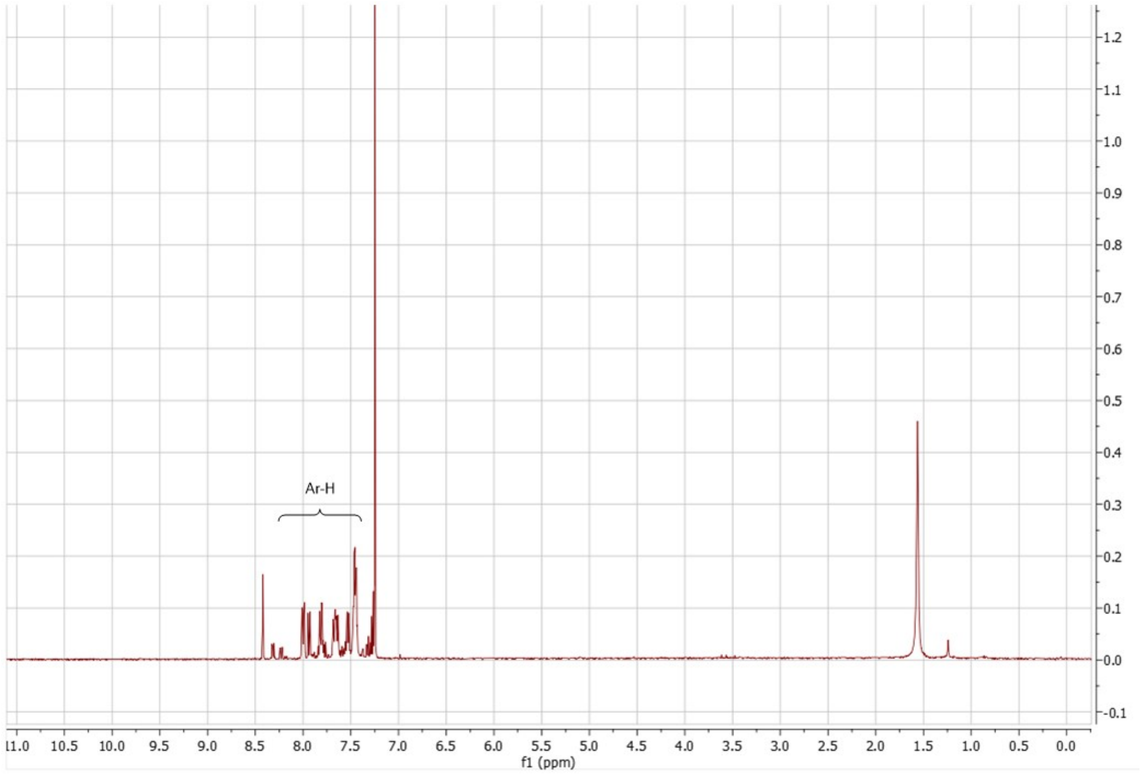

*Figure 13: ^{1}H NMR Spectrum of Polymer (**1A**)*

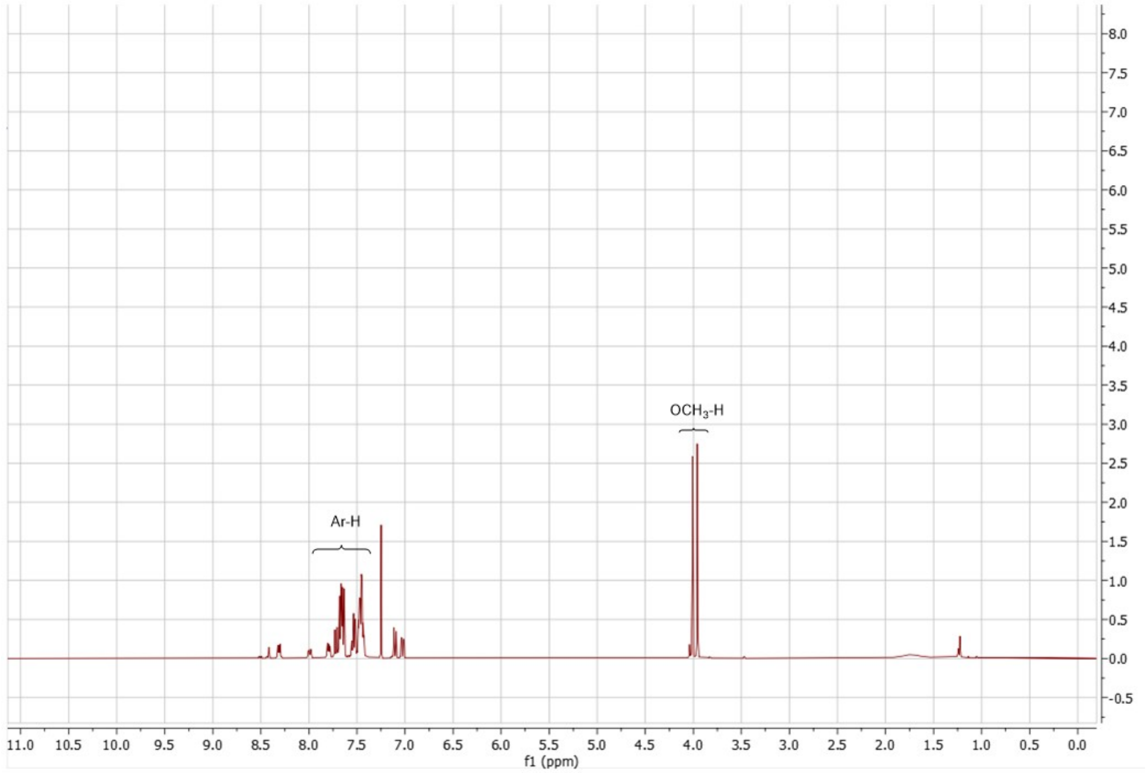

*Figure 14: ^{1}H NMR Spectrum of Polymer (**2A**)*

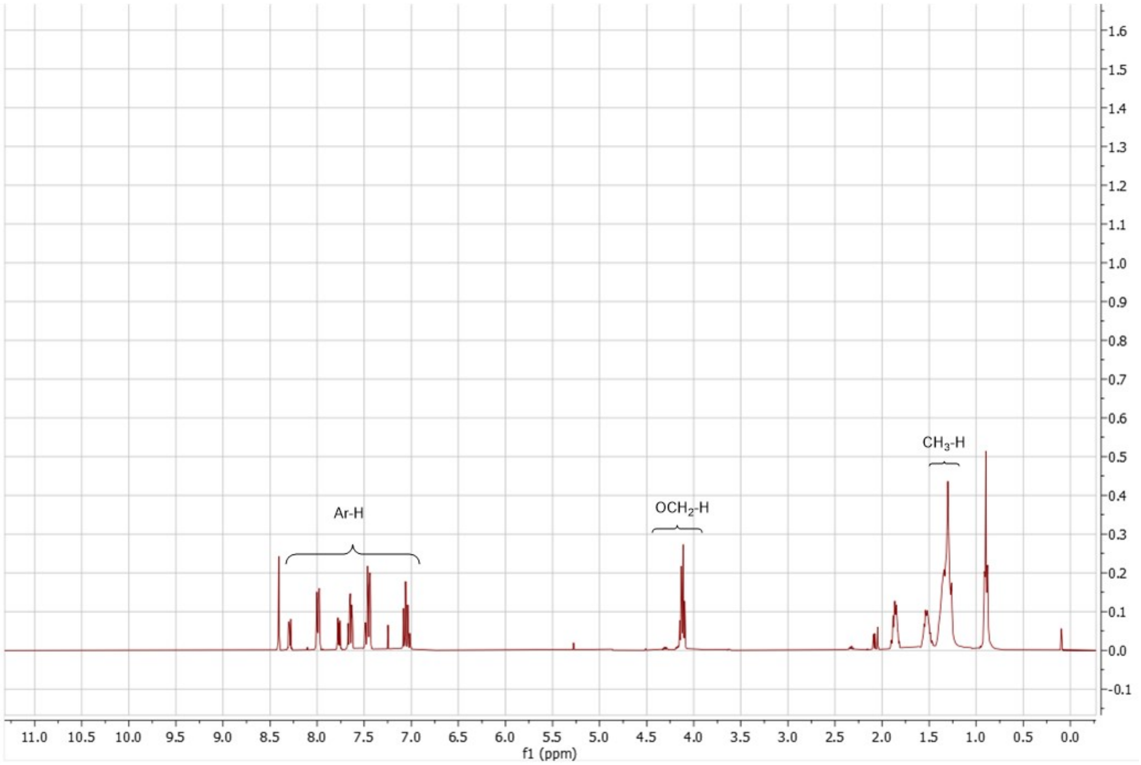

*Figure 15: ^{1}H NMR Spectrum of Polymer (**3A**)*

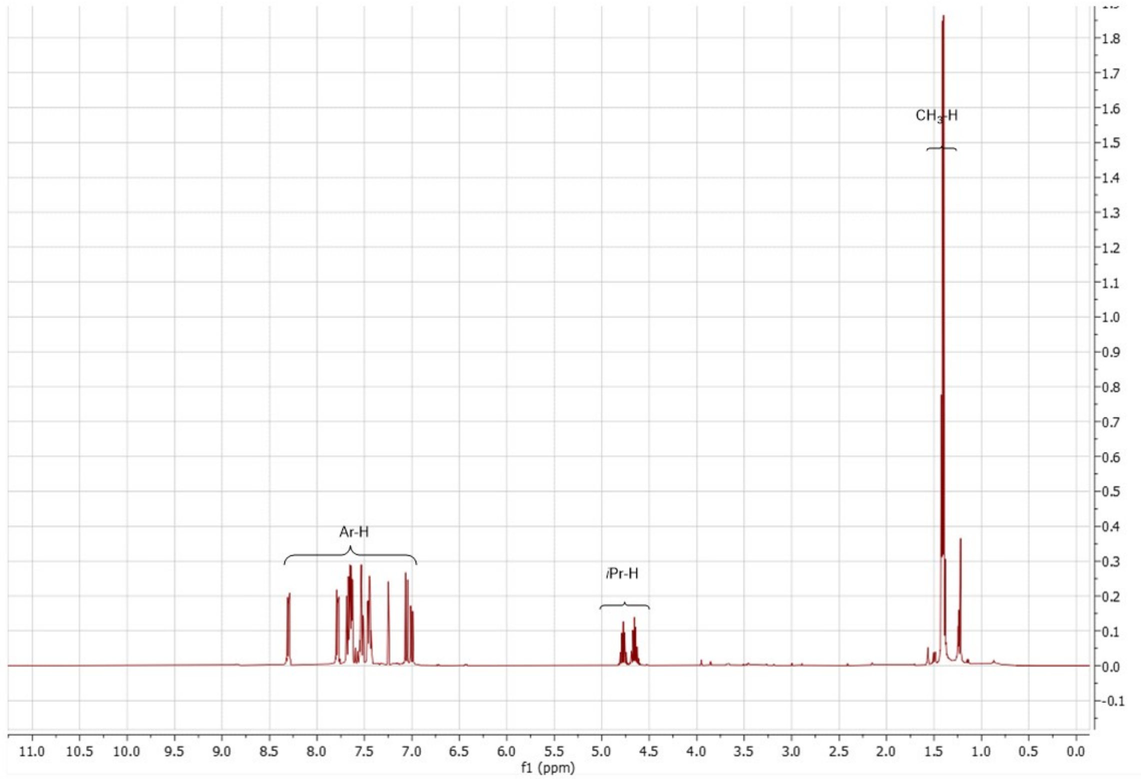

*Figure 16: ^{1}H NMR Spectrum of Polymer (**4A**)*

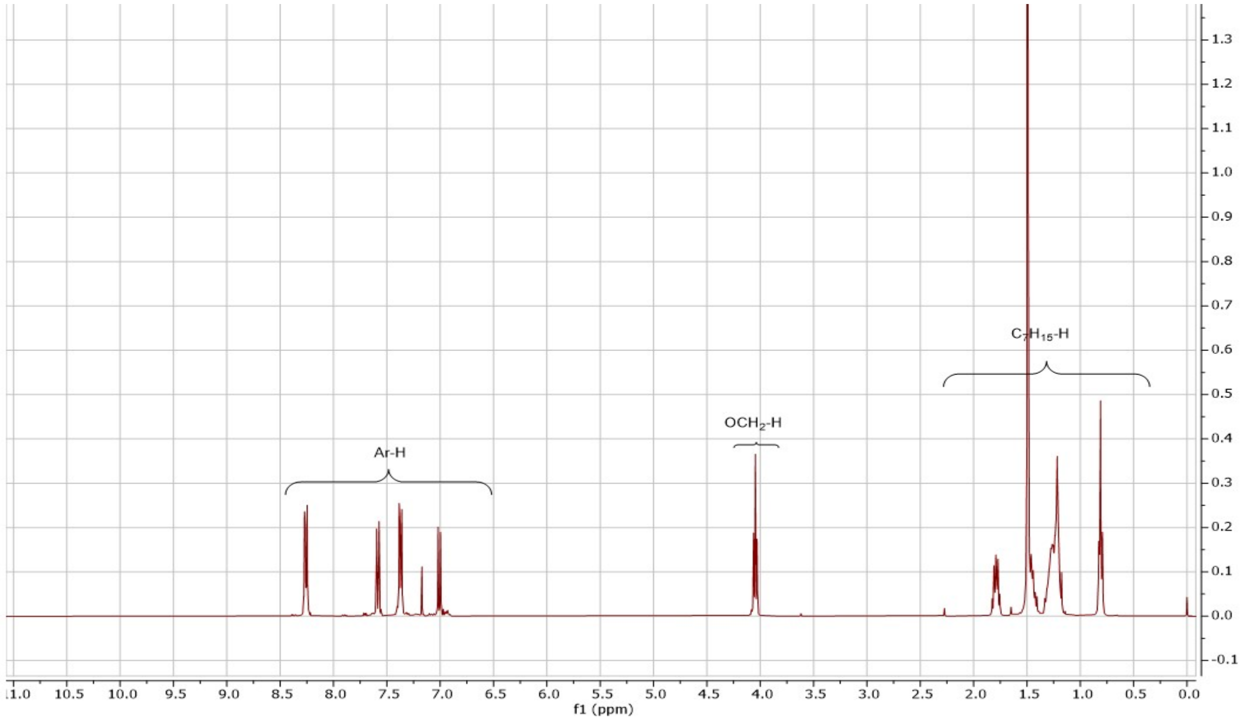

*Figure 17: ^{1}H NMR Spectrum of Polymer (**5A**)*

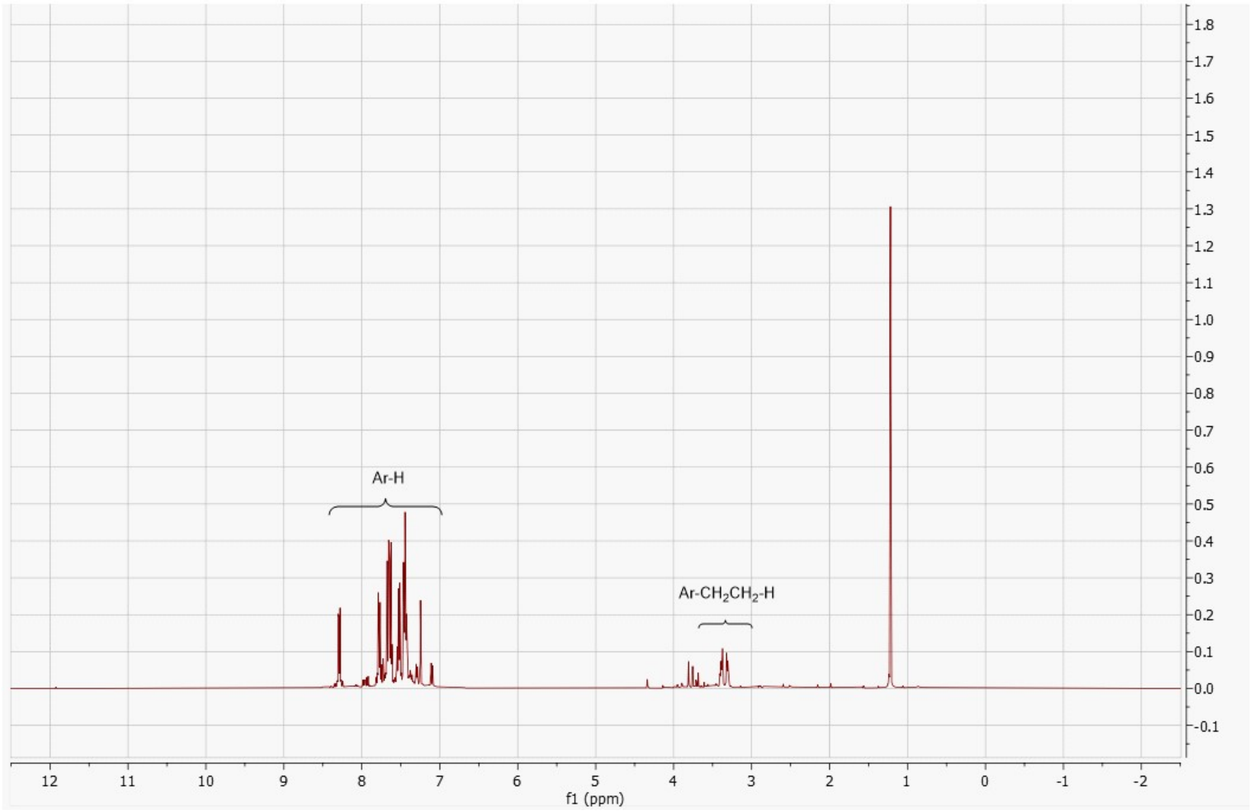

*Figure 18: ^{1}H NMR Spectrum of Polymer (**6A**)*

4. GPC Spectrum of polymers

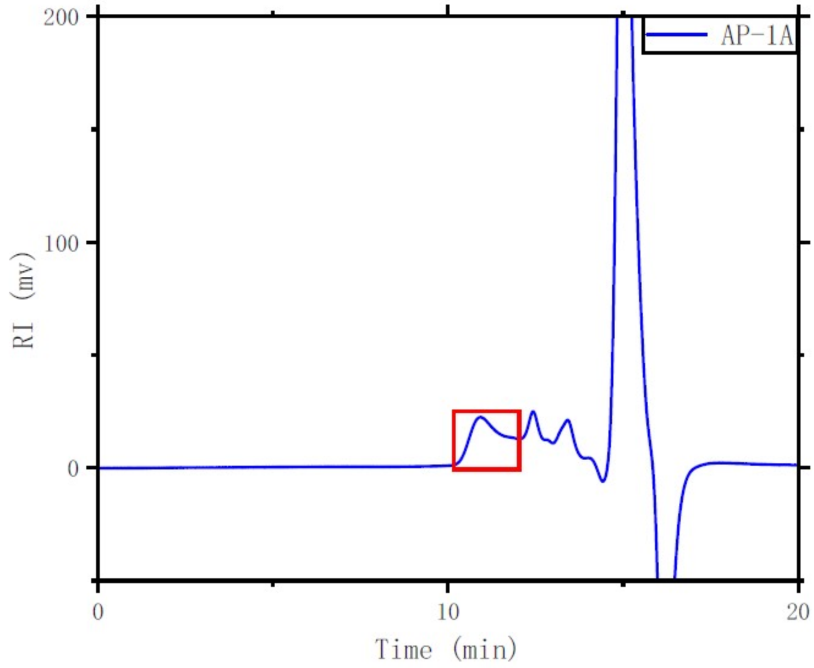

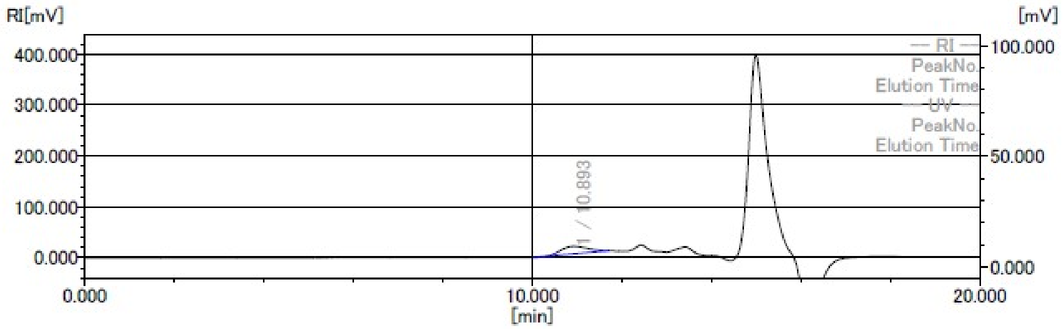

<Result of Molecular Weight Calculation>(RI)
Peak1Base Peak

	[min]	[mV]	[mol]
Peak Start	10.053	1.043	23789
Peak Top	10.893	22.387	7685
Peak End	11.698	13.716	2602
Height[mV]			14.873
Area[mV s]			619.160
Area%[%]			100.000
[Eta]			7642.05349

Mn	6681
Mw	7642
Mz	8719
Mz+1	9944
Mv	7642
Mp	7686
Mz/Mw	1.141
Mw/Mn	1.144
Mz+1/Mw	1.301

Figure 19: Gel permeation chromatography (GPC) Spectrum of Polymer 1A

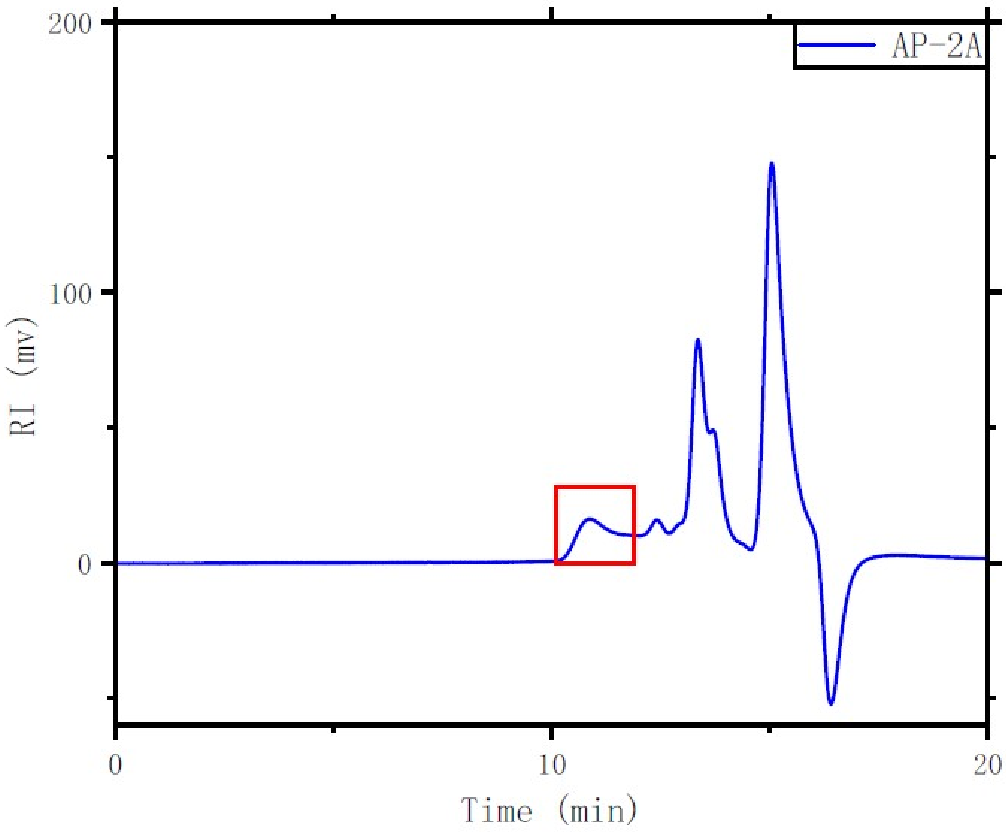

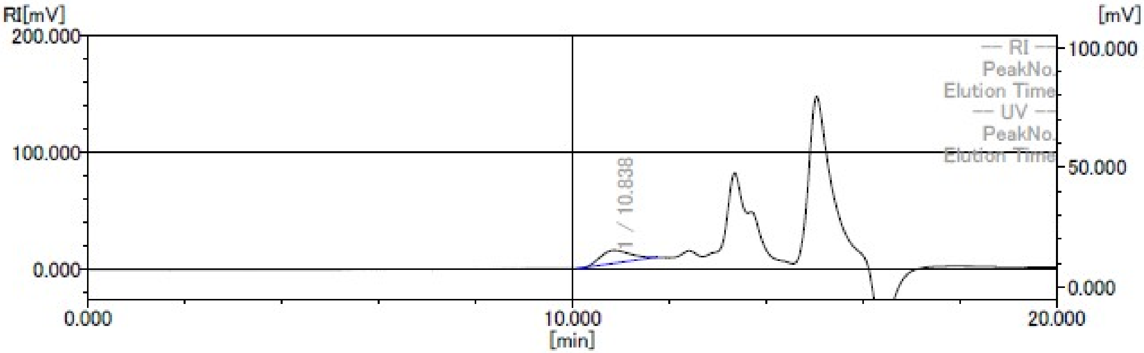

<Result of Molecular Weight Calculation>(RI)

Peak1Base Peak

	[min]	[mV]	[mol]		
Peak Start	10.123	0.837	21651	Mn	7006
Peak Top	10.838	16.195	8275	Mw	8050
Peak End	11.733	10.415	2483	Mz	9085
				Mz+1	10059
				Mv	8050
Height[mV]			11.104	Mp	8276
Area[mV s]			475.235	Mz/Mw	1.129
Area%[%]			100.000	Mw/Mn	1.149
[Eta]			8050.23872	Mz+1/Mw	1.250

Figure 20: Gel permeation chromatography (GPC) Spectrum of Polymer 2A

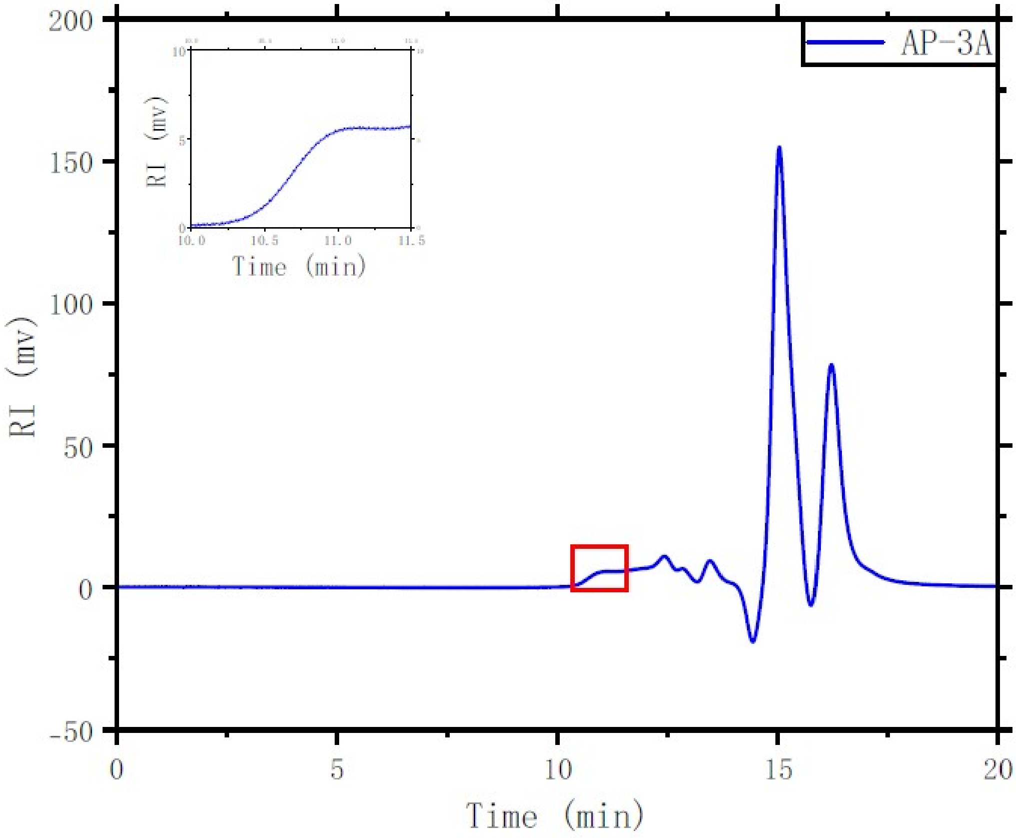

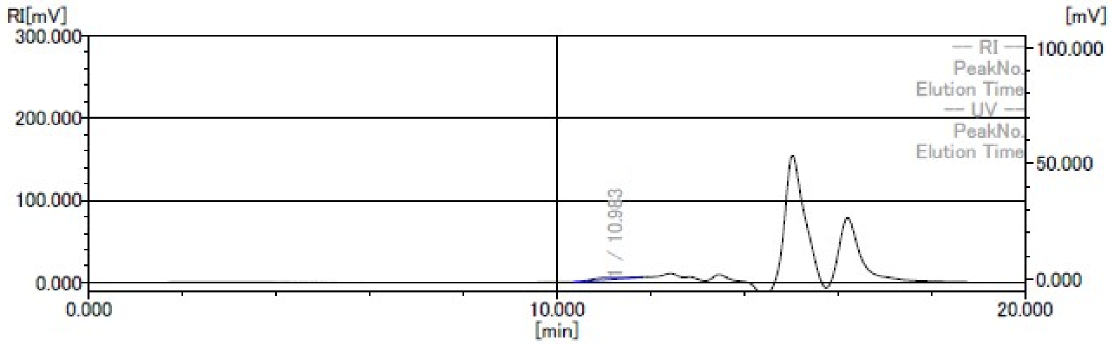

<Result of Molecular Weight Calculation>(RI)
Peak1Base Peak

	[min]	[mV]	[mol]		
Peak Start	10.368	0.606	15573	Mn	5891
Peak Top	10.983	5.505	6809	Mw	6677
Peak End	11.803	6.410	2259	Mz	7433
				Mz+1	8133
				Mv	6677
Height[mV]			2.412	Mp	6810
Area[mV s]			92.586	Mz/Mw	1.113
Area%[%]			100.000	Mw/Mn	1.133
[Eta]			6677.02317	Mz+1/Mw	1.218

Figure 21: Gel permeation chromatography (GPC) Spectrum of Polymer 3A

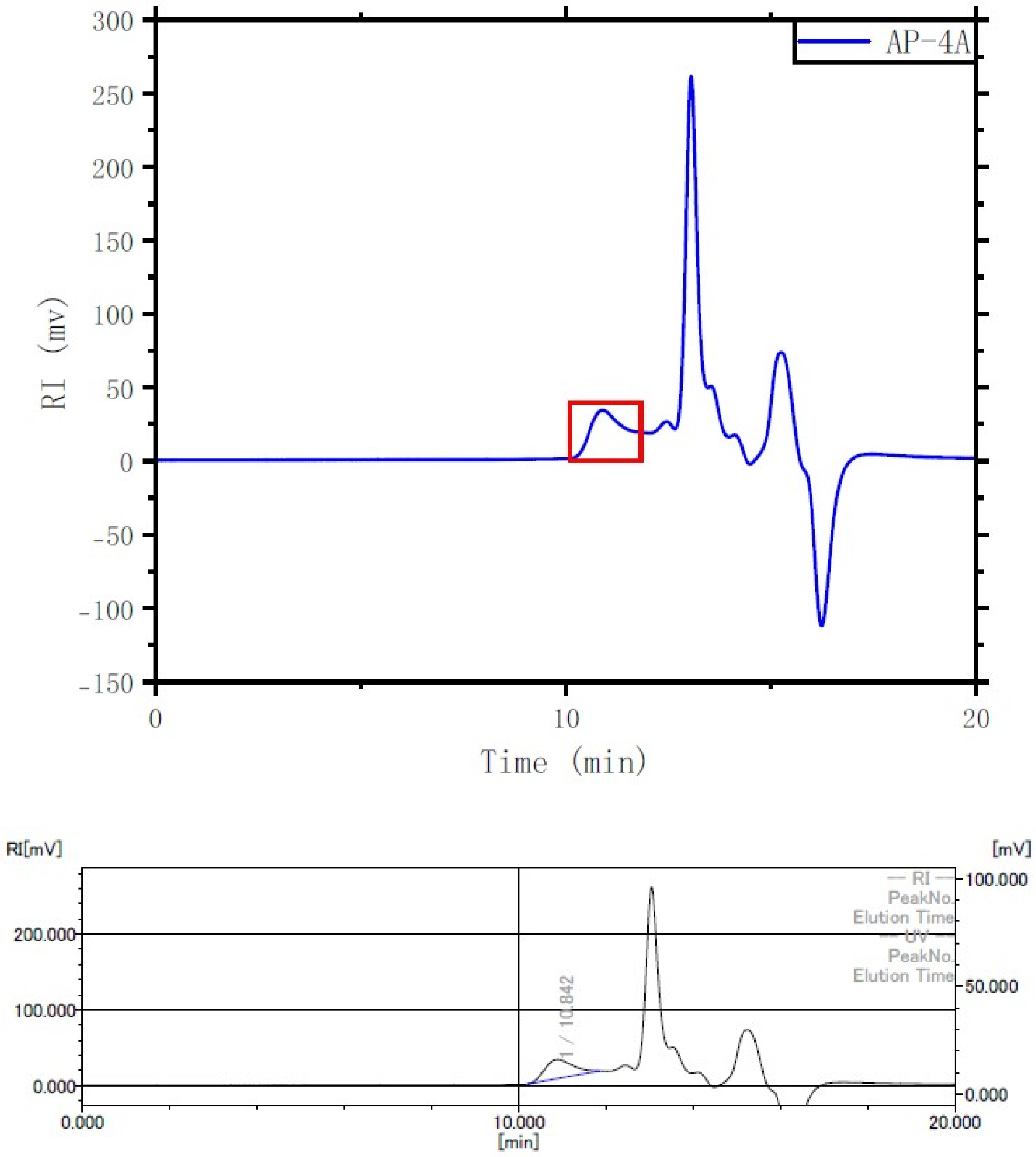

<Result of Molecular Weight Calculation>(RI)
Peak1Base Peak

	[min]	[mV]	[mol]
Peak Start	10.210	2.452	19269
Peak Top	10.842	34.277	8238
Peak End	11.857	19.537	2103
Height[mV]			25.271
Area[mV s]			1130.459
Area%[%]			100.000
[Eta]			7638.76231

Mn	6533
Mw	7639
Mz	8710
Mz+1	9689
Mv	7639
Mp	8239
Mz/Mw	1.140
Mw/Mn	1.169
Mz+1/Mw	1.268

Figure 22: Gel permeation chromatography (GPC) Spectrum of Polymer 4A

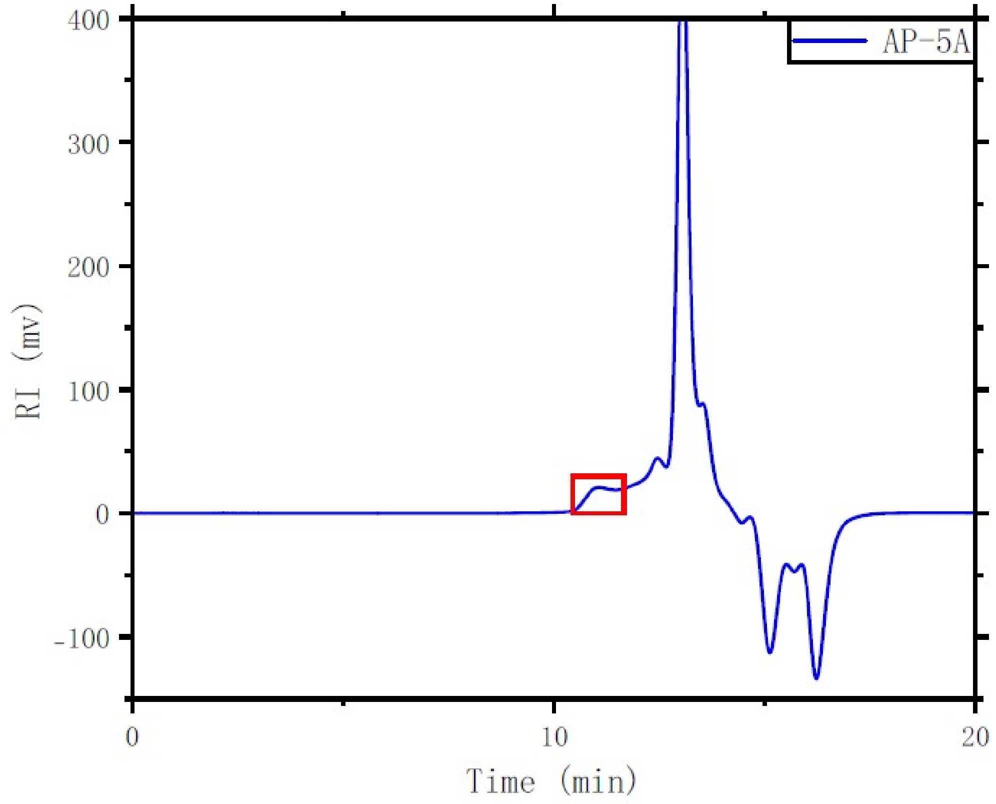

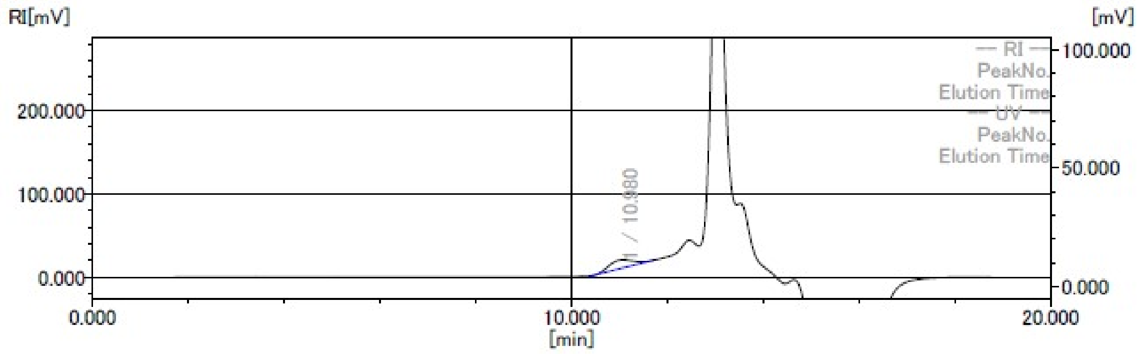

<Result of Molecular Weight Calculation>(RI)
Peak1Base Peak

	[min]	[mV]	[mol]
Peak Start	10.385	1.456	15227
Peak Top	10.980	20.291	6840
Peak End	11.717	20.452	2539
Height[mV]			10.347
Area[mV s]			349.261
Area%[%]			100.000
[Eta]			6667.87120

Mn	6126
Mw	6668
Mz	7214
Mz+1	7754
Mv	6668
Mp	6840
Mz/Mw	1.082
Mw/Mn	1.088
Mz+1/Mw	1.163

Figure 23: Gel permeation chromatography (GPC) Spectrum of Polymer 5A

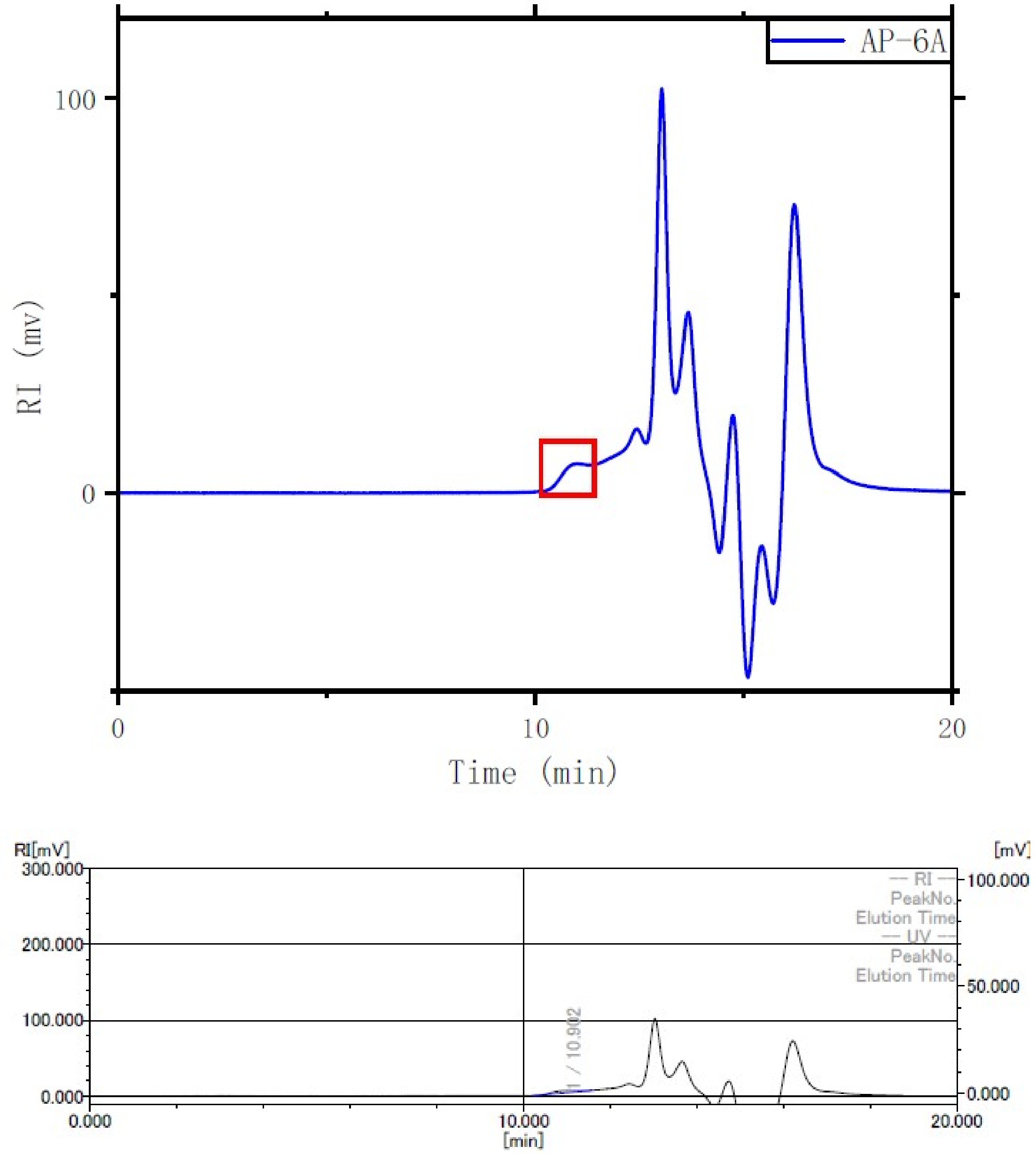

<Result of Molecular Weight Calculation>(RI)
Peak1Base Peak

	[min]	[mV]	[mol]		
Peak Start	10.228	0.606	18799	Mn	7171
Peak Top	10.902	7.242	7600	Mw	7872
Peak End	11.577	7.697	3065	Mz	8643
				Mz+1	9494
				Mv	7872
Height[mV]			3.095	Mp	7600
Area[mV s]			106.649	Mz/Mw	1.098
Area%[%]			100.000	Mw/Mn	1.098
[Eta]			7871.52771	Mz+1/Mw	1.206

Figure 24: Gel permeation chromatography (GPC) Spectrum of Polymer 6

References

1. Liu, K., Lalancette, R. A., & Jakle, F. B. (2017). B–N Lewis pair functionalization of anthracene: structural dynamics, optoelectronic properties, and O2 sensitization. . *Journal of the American Chemical Society* , 139, 18170–18173.

2. Tang, Y., Jin, S., Zhang, S., Wu, G.-Z., Wang, J.-Y., Xu, T., . . . others. (2022). Multilayer 3D chiral folding polymers and their asymmetric catalytic assembly. . *Research* .

3. Tang, Y., Zhang, S., Xu, T., Yuan, Q., Wang, J.-Y., Jin, S., . . . Chen, D. (2022). Aggregation-induced polarization (AIP): Optical rotation amplification and adjustment of chiral aggregates of folding oligomers and polymers. . *Frontiers in Chemistry*, 169, 111139.

4. Wu, G., Liu, Y., Yang, Z., Ma, L., Tang, Y., Zhao, X., . . . others. (2021). Triple-columned and multiple-layered 3D polymers: Design, syn-thesis, aggregation-induced emission (AIE), and computational study. . *Research* .

5. Xiao Liu, Q. Y.-l. (2015). *ARobust Microfluidic Device for the Synthesis and Crystal Growth of Organometallic Polymers with Highly Organized Structures.* Wiley.
